# Supplementary material for: Are socioenvironmental factors associated with psychotic symptoms in people with first-episode psychosis? A cross-sectional study of a West London clinical sample
Source: BMJ Open. 2019 Sep 18;9(9):e030448. doi: 10.1136/bmjopen-2019-030448 (PMC6756588; doi:10.1136/bmjopen-2019-030448)
Supplement: Supplementary data [file bmjopen-2019-030448supp001.pdf]

Supplementary Table 1 Key statistics for neighbourhood level variables (N=113 unique neighbourhoods included in the analysis). Statistics provided include the number of entries (N), the median and the interquartile range (IQR). The same data are also shown for all neighbourhoods in England (N=7932) for comparison, or else, all neighbourhoods with more than one composite LSOA (N=6685) where this was required to calculate an index (e.g. GINI-ID). Population density=general population density in people per hectare; ID=income deprivation; IMD=index of multiple deprivation; GINI-ID=Gini coefficient based on income deprivation; GINI-IMD=Gini coefficient based on index of multiple deprivation; SFI=social fragmentation index; SCI=social cohesion index; IDS-BME=index of dissimilarity for BME versus white populations; DEN-BME=BME ethnic density (people per hectare).

	Study data		National data	
	N	Median (IQR)	N	Median (IQR)
Pop density	113	63.91 (40.45-86.68)	7932	12.01 (0.02-236.05)
ID	113	11 (7.09-18)	7932	8.66 (1.03-61.71)
IMD	113	16.65 (10.2-24.33)	7932	14.68 (1.1-79.81)
GINI-ID	113	0.24 (0.18-0.3)	6685	0.21 (0-0.57)
GINI-IMD	113	0.17 (0.12-0.21)	6685	0.17 (0-0.65)
SFI	113	5.1 (1.76-7.69)	7932	-0.83 (-6.7-22.48)
SCI	113	33 (28.45-38.05)	7932	33.3
IDS-BME	113	0.13 (0.11-0.19)	6685	0.21 (0-0.79)
DEN-BME	113	0.17 (0.1-0.28)	7932	0.01 (0-0.86)

Supplementary Table 2 Spearman's correlations between neighbourhood level variables (n=113 unique neighbourhoods). Provided statistics include Spearman's Rho and p value (in brackets). Significant correlations at an alpha of 0.002 (corrected for 21 comparisons) are shown in bold. POP DENS=population density; IMD=index of multiple deprivation; GINI-IMD=Gini coefficient calculated for the index of multiple deprivation; SFI=social fragmentation index; SCI=social cohesion index (voter turnout); IDS-BME=index of dissimilarity for BME versus white populations; DEN-BME=BME ethnic density.

	IMD	GINI -IMD	SFI	SCI	IDS-BME	DEN-BME
POP DENS	**0.38 (<0.001)**	**-0.31 (<0.001)**	**0.65 (<0.001)**	**-0.42 (<0.001)**	0.24 (<0.01)	**0.31 (<0.001)**
IMD		**-0.38 (<0.001)**	**0.32 (<0.001)**	**-0.62 (<0.001)**	**0.29 (<0.01)**	**0.75 (<0.001)**
GINI-IMD		-	**-0.32 (<0.001)**	**0.45 (<0.001)**	0.05 (0.59)	**-0.31 (<0.001)**
SFI			-	**-0.41 (<0.001)**	0.09 (0.33)	0.05 (0.6)
SCI				-	-0.18 (0.06)	**-0.59 (<0.001)**
IDS-BME					-	**0.3 (<0.01)**

Supplementary Table 3 Negative symptoms, derived from second- order principle components analysis of individual item scores, regressed on predictor variables. The top line of the table indicates a random effects only model. Columns three and four show the results of a series of univariate analyses, examining the effects of adding individual predictors to a model that controls for core demographics only (age, gender and socioeconomic status). Model one shows the results of a multivariate analysis that includes all variables selected for inclusion. Model two is identical to model one except that other symptoms (positive and disorganisation symptoms) are also controlled for. Neighbourhood level variables are in italics. Significant p values are in bold.

		Univariate (controlling for basic demographics only)					Model 1 – Multivariate (basic)		Model 2 – Multivariate (controlling for other Sxs also)	
		Fixed part of model		Random part of model			Fixed part of the model		Fixed part of the model	
Predictor	Level	Coefficient (95% CIs)	Wald p value	Neighbourhood level variance	Chi-squared (1 df)	Chi-squared p value	Coefficient (95% CIs)	Wald p value	Coefficient (95% CIs)	Wald p value
Random only		-	-	0	0	1	-	-	-	-
Age		0.01 (-0.01, 0.02)	0.45	0	0	1	0.01 (-0.01, 0.02)	0.33	0.01 (-0.01, 0.02)	0.37
Gender	Female	-0.32 (-0.56, -0.08)	**<0.01**	0	0	1	-0.33 (-0.56, -0.09)	**<0.01**	-0.36 (-0.59, -0.12)	**<0.01**
NS-SEC	Managerial	-0.51 (-1.01, -0.01)	0.05	0	0	1	-0.47 (-0.97, 0.03)	0.06	-0.49 (-0.98, 0)	0.05
	Intermediate	-0.26 (-0.71, 0.19)	0.26	0	0	1	-0.2 (-0.65, 0.24)	0.37	-0.23 (-0.67, 0.22)	0.32
	Routine	-0.33 (-0.64, -0.02)	**0.03**	0	0	1	-0.31 (-0.61, -0.01)	**<0.05**	-0.35 (-0.65, -0.04)	**0.03**
	Student	0.08 (-0.24, 0.41)	0.61	0	0	1	0.12 (-0.2, 0.44)	0.46	0.09 (-0.23, 0.4)	0.59
Pop Den		0 (0, 0)	0.35	0	0	1	-	-	-	-
ID		-0.01 (-0.02, 0.01)	0.25	0	0	1	-	-	-	-
IMD		-0.01 (-0.02, 0.01)	0.39	0	0	1	-	-	-	-
GINI-ID		-1.54 (-2.76, -0.33)	**0.01**	0	0	1	-1.54 (-2.76, -0.33)	**0.01**	-1.66 (-2.86, -0.46)	**<0.01**
GINI-IMD		-1.42 (-2.93, 0.09)	0.07	0	0	1	-	-	**-**	**-**
SFI		-0.02 (-0.05, 0.01)	0.24	0	0	1	-	-	-	-
SCI		0 (-0.02, 0.02)	0.77	0	0	1	-	-	-	-
IDS-BME		-1.51 (-3.35, 0.33)	0.11	0	0	1	-	-	-	-
DEN-BME		0.33 (-0.27, 0.93)	0.28	0	0	1	-	-	-	-

Supplementary Table 4 Positive symptoms, derived from second- order principle components analysis of individual item scores, regressed on predictor variables. The top line of the table indicates a random effects only model. Columns three and four show the results of a series of univariate analyses, examining the effects of adding individual predictors to a model that controls for core demographics only (age, gender and socioeconomic status). Model one shows the results of a multivariate analysis that includes all variables selected for inclusion. Model two is identical to model one except that other symptoms (negative and disorganisation symptoms) are also controlled for. Neighbourhood level variables are in italics. Significant p values are in bold.

		Univariate (controlling for basic demographics only)					Model 1 –Multivariate (basic)		Model 2 –Multivariate (controlling for other Sxs also)	
		Fixed part of model		Random part of model			Fixed part of the model		Fixed part of the model	
Predictor	Level	Coefficient (95% CIs)	Wald p value	Neighbourhood level variance	Chi-squared (1 df)	Chi-squared p value	Coefficient (95% CIs)	Wald p value	Coefficient (95% CIs)	Wald p value
Random only		-	-	0	0	1	-	-	-	-
Age		-0.01 (-0.02, 0)	0.11	0	0	1	-0.01 (-0.02, 0)	0.12	-0.01 (-0.02, 0)	0.12
Gender	Female	0.04 (-0.2, 0.28)	0.75	0	0	1	0.05 (-0.19, 0.28)	0.7	0.06 (-0.18, 0.3)	0.63
NS-SEC	Managerial	-0.04 (-0.55, 0.46)	0.86	0	0	1	0.01 (-0.49, 0.51)	0.96	0.03 (-0.47, 0.53)	0.91
	Intermediate	0.47 (0.01, 0.92)	**0.04**	0	0	1	0.49 (0.04, 0.93)	**0.03**	0.49 (0.05, 0.94)	**0.03**
	Routine	0.15 (-0.16, 0.46)	0.35	0	0	1	0.14 (-0.16, 0.45)	0.35	0.16 (-0.15, 0.47)	0.32
	Student	0.11 (-0.22, 0.43)	0.51	0	0	1	0.07 (-0.25, 0.39)	0.68	0.07 (-0.25, 0.39)	0.67
Pop Den		0 (0, 0)	0.49	0	0	1	-	-	-	-
ID		-0.01 (-0.02, 0.01)	0.27	0	0	1	-	-	-	-
IMD		-0.01 (-0.02, 0)	0.22	0	0	1	-	-	-	-
GINI-ID		0.42 (-0.81, 1.65)	0.5	0	0	1	-	-	-	-
GINI-IMD		0.58 (-0.94, 2.1)	0.46	0	0	1	-	-	-	-
SFI		0.01 (-0.02, 0.04)	0.72	0	0	1	-	-	-	-
SCI		0.02 (0, 0.04)	0.08	0	0	1	-	-	-	-
IDS-BME		-2.36 (-4.2, -0.52)	**0.01**	0	0	1	-2.36 (-4.2, -0.52)	**0.01**	-2.32 (-4.17, -0.48)	**0.01**
DEN-BME		-0.55 (-1.15, 0.05)	0.07	0	0	1	-	-	-	-

Supplementary Table 5 Disorganisation symptoms, derived from second- order principle components analysis of individual item scores, regressed on predictor variables. The top line of the table indicates a random effects only model. Columns three and four show the results of a series of univariate analyses, examining the effects of adding individual predictors to a model that controls for core demographics only (age, gender and socioeconomic status). Model one shows the results of a multivariate analysis that includes all variables selected for inclusion. Model two is identical to model one except that other symptoms (negative and positive symptoms) are also controlled for. Neighbourhood level variables are in italics. Significant p values are in bold.

		Univariate (controlling for basic demographics only)					Model 1 –Multivariate (basic)		Model 2 –Multivariate (controlling for other Sxs also)	
		Fixed part of model		Random part of model			Fixed part of the model		Fixed part of the model	
Predictor	Level	Coefficient (95% CIs)	Wald p value	Neighbourhood level variance	Chi-squared (1 df)	Chi-squared p value	Coefficient (95% CIs)	Wald p value	Coefficient (95% CIs)	Wald p value
Random only		-	-	0.45	0.01	0.46	-	-	-	-
Age		-0.01 (-0.02, 0.01)	0.27	1.45	0.01	0.38	-0.01 (-0.02, 0.01)	0.27	-0.01 (-0.02, 0.01)	0.33
Gender	*Female*	-0.18 (-0.42, 0.06)	0.14	1.45	0.01	0.38	-0.18 (-0.42, 0.06)	0.14	-0.23 (-0.47, 0.01)	0.06
NS-SEC	*Managerial*	-0.15 (-0.65, 0.36)	0.57	1.45	0.01	0.38	-0.15 (-0.65, 0.36)	0.57	-0.22 (-0.73, 0.29)	0.39
	Intermediate	-0.04 (-0.5, 0.42)	0.86	1.45	0.01	0.38	-0.04 (-0.5, 0.42)	0.86	-0.08 (-0.54, 0.37)	0.72
	Routine	-0.21 (-0.52, 0.1)	0.19	1.45	0.01	0.38	-0.21 (-0.52, 0.1)	0.19	-0.26 (-0.57, 0.05)	0.1
	Student	-0.22 (-0.54, 0.11)	0.19	1.45	0.01	0.38	-0.22 (-0.54, 0.11)	0.19	-0.21 (-0.53, 0.12)	0.21
Pop Den		0 (0, 0)	0.53	1.1	0.05	0.41	-	-	-	-
ID		0.01 (-0.01, 0.02)	0.31	1.28	0.07	0.39	-	-		
IMD		0.01 (0, 0.02)	0.25	1.1	0.05	0.41	-	-	-	-
GINI-ID		-0.65 (-1.9, 0.6)	0.23	0.85	0.03	0.43	-	-	-	-
GINI-IMD		-0.84 (-2.39, 0.71)	0.29	0.86	0.03	0.43	-	-	-	-
SFI		0 (-0.03, 0.04)	0.76	1.41	0.09	0.38	-	-	-	-
SCI		-0.01 (-0.03, 0)	0.14	0.76	0.03	0.44	-	-	-	-
IDS-BME		0.29 (-1.62, 2.19)	0.77	1.37	0.08	0.39	-	-	-	-
DEN-BME		0.23 (-0.38, 0.85)	0.46	0.87	0.03	0.43	-	-	-	-

Supplementary Table 6 Multi-level modelling of paranoid delusions and global hallucinations. Relevant scores were regressed on predictor variables using multi-level logistic analyses. Significant p values are in bold.

		Persecutory delusions		Global hallucinations	
		Fixed part of the model		Fixed part of the model	
Predictor	Level	Odds ratio (95% CIs)	Wald p value	Odds ratio (95% CIs)	Wald p value
Age		1.03 (1, 1.06)	0.09	0.99 (0.96, 1.02)	0.36
Gender	Female	1.01 (0.59, 1.72)	0.97	0.86 (0.52, 1.44)	0.57
NS-SEC	Managerial	0.22 (0.07, 0.66)	**<0.01**	1.58 (0.51, 4.9)	0.43
	Intermediate	0.91 (0.32, 2.59)	0.86	1 (0.38, 2.59)	1
	Routine	0.75 (0.38, 1.46)	0.39	1.04 (0.55, 2)	0.9
	Student	0.72 (0.36, 1.44)	0.36	2.11 (0.97, 4.63)	0.06
