# Supplementary material for: Are socioenvironmental factors associated with psychotic symptoms in people with first-episode psychosis? A cross-sectional study of a West London clinical sample
Source: BMJ Open. 2019 Sep 18;9(9):e030448. doi: 10.1136/bmjopen-2019-030448 (PMC6756588; doi:10.1136/bmjopen-2019-030448)
Supplement: Author's manuscript [file bmjopen-2019-030448.draft_revisions.pdf]

BMJ Open

BMJ Open is committed to open peer review. As part of this commitment we make the peer review history of every article we publish publicly available.

When an article is published we post the peer reviewers' comments and the authors' responses online. We also post the versions of the paper that were used during peer review. These are the versions that the peer review comments apply to.

The versions of the paper that follow are the versions that were submitted during the peer review process. They are not the versions of record or the final published versions. They should not be cited or distributed as the published version of this manuscript.

BMJ Open is an open access journal and the full, final, typeset and author-corrected version of record of the manuscript is available on our site with no access controls, subscription charges or pay-per-view fees (http://bmjopen.bmj.com).

If you have any questions on BMJ Open's open peer review process please email info.bmjopen@bmj.com

BMJ Open

Socio-environmental Predictors of Psychotic Symptoms in a West London Clinic Sample of People with First-episode Psychosis

Journal:	*BMJ Open*
Manuscript ID	bmjopen-2019-030448
Article Type:	Research
Date Submitted by the Author:	14-Mar-2019

Complete List of Authors:	Tibber, Marc; University College London, Department of Clinical, Educational and Health Psychology;<br>Kirkbride, James; University College London, Division of Psychiatry<br>Mutsatsa, Stanley ; School of Health Sciences, City, University of London<br>Harrison, Isobel ; University College London, Division of Psychiatry<br>Barnes, Thomas; Imperial College London, Centre for Psychiatry<br>Joyce, Eileen; University College London, Institute of Neurology<br>Huddy, Vyv; University of Sheffield, Clinical Psychology Unit, Department of Psychology

Keywords:	Schizophrenia & psychotic disorders < PSYCHIATRY, EPIDEMIOLOGY, Adult psychiatry < PSYCHIATRY

SCHOLARONE™
Manuscripts

Socio-environmental Predictors of Psychotic Symptoms in a West London Clinic Sample of People with First-episode Psychosis

Marc S. Tibber[a], James B. Kirkbride[b], Stanley Mutsatsa[c], Isobel Harrison[b], Thomas R. E. Barnes[d], Eileen M. Joyce[e] ,Vyv Huddy[f]

[a]Department of Clinical, Educational and Health Psychology, UCL, London, UK; [b]Division of Psychiatry, UCL, London, UK; [c]School of Health Sciences, City, University of London, UK; [d]Centre for Psychiatry, Imperial College London, UK; [e]UCL Institute of Neurology, London, UK; [f]Clinical Psychology Unit, Department of Psychology, University of Sheffield, UK.

Corresponding author:
Marc Tibber
Email: m.tibber@ucl.ac.uk
0 44 (0)20 7679 1673.

For peer review only

For peer review only - http://bmjopen.bmj.com/site/about/guidelines.xhtml

Abstract

Objectives: To determine whether neighbourhood level socio-environmental factors including deprivation and inequality predict variance in psychotic symptoms after controlling for individual-level demographics.

Design: A cross-sectional design involving secondary analysis of a pre-existing data-set was used.

Setting: Data were originally collected from secondary care services within the UK boroughs of Ealing, Hammersmith and Fulham, Wandsworth, Kingston, Richmond, Merton, Sutton and Hounslow as part of the West London First-Episode Psychosis study.

Participants: Complete case analyses were undertaken on 319 participants who met the following inclusion criteria: aged 16 years or over, resident in the study's catchment area, experiencing a first psychotic episode, with fewer than 12 weeks exposure to antipsychotic medication and sufficient command of English to facilitate assessment.

Outcome measures: Symptom dimension scores, derived from principle components analyses of the SAPS and SANS, were regressed on neighbourhood level predictors, including population density, income deprivation, income inequality, social fragmentation, social cohesion, ethnic density and ethnic fragmentation, using multi-level regression.

Results: Higher income inequality was associated with lower negative symptom scores (coefficient=-1.66, CI=-2.86, -0.46, p<0.01) and higher levels of ethnic segregation were associated with lower positive symptom scores (coefficient=-2.32, CI=-4.17, -0.48, p=0.01) after adjustment for covariates.

Conclusions: These findings provide further evidence that particular characteristics of the environment may be linked to specific symptom clusters in psychosis.

For peer review only

Longitudinal studies are required to begin to tease apart the underlying mechanisms involved as well as the causal direction of such associations.

Strengths and limitations of the study:

- This study goes beyond incidence / prevalence level research to explore the environmental predictors of psychosis at the resolution of symptoms and symptom clusters.
- This symptom level approach has the potential to further our understanding of underlying etiological mechanisms and is in line with current developments in clinical and research practice.
- The study uses a multi-level analysis approach that is perfectly suited to the nested / hierarchical structure of the data.
- The main limitation of the study is its cross-sectional design, which precludes inferences about causality.

Funding: The West London Study of First Episode Psychosis was funded by a Wellcome Trust Programme grant to Eileen Joyce, Thomas Barnes, Maria Ron and Gareth Barker (grant number 064607/Z/01/Z). James Kirkbride was supported by a Sir Henry Dale Fellowship, jointly funded by the Wellcome Trust and the Royal Society (Grant no. 101272/Z/13/Z). Eileen Joyce and James Kirkbride were supported by the National Institute for Health Research University College London Hospitals Biomedical Research Centre. On behalf of all authors, the corresponding author states that there is no conflict of interest.

For peer review only - http://bmjopen.bmj.com/site/about/guidelines.xhtml

Background

Epidemiological studies exploring geographical variation in the incidence of psychotic disorders have highlighted various characteristics of environments that are typically associated with an increased risk including high population density [1], urbanicity [2], income deprivation [3], income inequality [1,4,5] and low social cohesion [6]. More recently, researchers have begun to investigate whether such risk factors are associated not with the *incidence* of psychosis, but with the *severity* of psychosis symptoms or symptom cluster scores. Underlying this approach is the notion that exposure to particular types of adverse environments and experiences may be causally linked to the development or exacerbation of specific psychological symptoms [7]. For example, it has been suggested that densely-populated, deprived, inner-city areas may be causally associated with the development and expression of positive symptoms, e.g. paranoid delusions and hallucinations [8,9]. It has been hypothesised that this association may be mediated, at least in part, by the increased sense of disempowerment, victimisation and social alienation that (arguably) characterise such neighbourhoods [10–12].

To date only a handful of studies have used an epidemiological approach to explore the association between socio-environmental adversity and specific psychosis symptoms or symptom clusters. Whilst these studies have typically reported associations between indices of environmental adversity and some form of positive symptom expression, associations with specific symptom items were not consistent across studies. For example, whilst Oher et al. [13] reported a significant association between neighbourhood level population density and hallucinations but *not* paranoia in first episode psychosis (FEP), Wickham et al. [14] found an inverse pattern of associations with neighbourhood level deprivation in the general population: the most deprived neighbourhoods were associated with increased paranoia but *not* hallucinations. In addition, reported associations between socio-environmental

For peer review only - http://bmjopen.bmj.com/site/about/guidelines.xhtml

adversity and symptom severity in these studies were not restricted to positive symptoms; associations were also seen with depressive symptoms [13,14]. (See [15,16] also).

It is unclear whether this variation -or arguably discrepancy- in findings is due to small effect sizes / chance, or instead, methodological differences between studies. For example, the studies described were undertaken on different population samples (clinical vs. non-clinical) at different levels of analysis (within a country at the level of neighbourhoods vs. across countries) using distinct indices of socio-environmental adversity (urbanicity, deprivation and/or inequality) and different symptom measures [13,14]. Nonetheless, the possibility that specific symptom clusters may be linked to defined socio-environmental risk factors suggests value in moving away from studies of diagnostic incidence towards a focus on the predictors of psychotic symptoms and symptom clusters. By virtue of their greater specificity symptom level analyses of this kind may be more informative as to the underlying aetiological mechanisms involved, as well as potential targets for intervention.

In a recent study of the symptom dimensions underlying psychotic disorders in a first episode psychosis (FEP) cohort in West London we have shown that symptoms were best characterised by positive, negative and disorganisation syndromes [17]. Here, we examined the extent to which these three symptom dimensions were predicted by neighbourhood level socio-environmental factors, including indices of deprivation, inequality and social capital. Given the extant literature in the field [13,14,16,18] we predicted that at the syndrome level, indices of socio-environmental adversity would be associated with higher levels of positive symptoms. Further, given the findings reported by Oher et al. [13] in their FEP sample (which most closely matches our own), at an item level of analysis we

For peer review only - http://bmjopen.bmj.com/site/about/guidelines.xhtml

predicted that socio-environmental adversity would predict hallucinations, but not paranoia.

Methods

Setting

A secondary analysis was undertaken on data originally collected as part of the prospective West London First-Episode Psychosis study (WLFEP) [19,20]. Participants presented to secondary care services within the boroughs of Ealing, Hammersmith and Fulham, Wandsworth, Kingston, Richmond, Merton, Sutton and Hounslow, between 1998 and 2006. Ethical approval was obtained from local ethics committees of all boroughs included and written informed consent was obtained (RREC 3006). The study was therefore undertaken in accordance with the ethical standards defined in the 1964 Declaration of Helsinki and subsequent amendments therein.

Participants

Participants included for participation were aged 16 years or over, experiencing a first psychotic episode, had received fewer than 12 weeks antipsychotic medication and spoke sufficient English to facilitate assessment. Potential participants were screened using the World Health Organization Psychosis Screen [21] and diagnosed according to DSM-III [22] and DSM-IV criteria [23] by two psychiatric research nurses using the diagnostic module of the Diagnostic Interview for Psychosis [24]. See [17,25] for full details.

Data collection

Information was obtained, with informed consent, from participants' clinical records and clinical interview. Data gathered included basic demographics and symptom scores as well as performance on a number of clinical, cognitive and

For peer review only - http://bmjopen.bmj.com/site/about/guidelines.xhtml

neuropsychological assessments. All researchers involved in data collection received training to a high standard in the administration of these measures. All data presented were gathered at baseline / initial recruitment to the study.

Measures

Symptom dimension scores

Each participant's symptoms were characterised by three symptom dimension scores, which captured the severity of their positive, negative and disorganisation symptoms. These were derived from a second-order principle components analysis (PCA) of participants' individual item scores on the Scale for the Assessment of Positive Symptoms (SAPS) and the revised version of the Scale for the Assessment of Negative Symptoms (SANS-R) [26]; see [17,27] for further details.

Individual level covariates

Basic demographic information including age, gender and place of residence were gathered. Ethnicity data were not recorded. In addition, participants were assigned to one of five socioeconomic categories based on their occupation using the National Statistics Socio-Economic Classification system (NS-SEC) [28].

Neighbourhood level exposures

Each participant's postcode was used to identify their small area neighbourhood, based on the Census Area Statistics (CAS) ward in which they lived at first contact using databases produced by the ONS [29]. For each ward -henceforth referred to as a neighbourhood- a number of indices of urbanicity, deprivation, inequality and social capital were identified; unless specified otherwise, all these data were obtained from the 2001 census [29].

For peer review only - http://bmjopen.bmj.com/site/about/guidelines.xhtml

Following the work of others [30], population density (measured in people per hectare) was used as a proxy for urbanicity.

Two indices of deprivation were included in the study: (i) income deprivation (ID): the percentage of individuals who were living in a household with an income of less than 60% of the median, and (ii) the Index of Multiple Deprivation (IMD), an aggregate measure of deprivation comprised of 37 indicators, obtained from The English Indices of Deprivation (2004). Since these data were available for nested geographical areas smaller than the CAS ward –lower layer super output areas (LSOA)- neighbourhood level indices were calculated as the sum of composite LSOA values, weighted by their population size, as described previously [1].

In addition, it was also possible to derive indices of *inequality,* i.e. the dispersion of deprivation. For both the ID and IMD, a corresponding Gini coefficient was calculated for each neighbourhood (GINI-ID and GINI-IMD), based on the distribution of deprivation across its composite LSOAs, as described previously [1]. A Gini coefficient of zero represents perfect equality, whereas Gini coefficients approaching one indicate maximum inequality.

To calculate an index of social fragmentation (SFI), a composite of four separate 2001 census measures (Z-transformed and summed) were used, as described previously [31,32]: (i) the percentage of people aged 16 years or over and single; (ii) the percentage of households that were single-occupancy; (iii) the percentage of households that were rented; and (iv) the percentage of people who were mobile in the 12 months prior to the census date. As a proxy for social cohesion, voter turnout during the 2002 local elections (percentage of the electorate who cast valid ballots) was obtained for each neighbourhood [33] as described previously [6,13].

For peer review only - http://bmjopen.bmj.com/site/about/guidelines.xhtml

Ethnic segregation was estimated using the Index of Dissimilarity (IDS) [6]. IDS was calculated at the neighbourhood level, measuring the extent to which white and black / minority / ethnic (BME) populations were segregated across Lower Output Areas (LSOAs) within each neighbourhood (IDS-BME). IDS scores ranged between 0 (no segregation) to 1 (total segregation). In addition, BME ethnic density was calculated (DEN-BME) as the proportion of BME individuals relative to the total population in each neighbourhood.

For peer review only

Statistical analyses

To determine the neighbourhood level predictors of psychotic symptoms, symptom dimension scores were regressed on predictors using multi-level linear regression. Prior to regression symptom dimension scores were transformed to minimize skew, using an optimal transformation (square-root, cube-root, logarithmic or inverse transformations) on the basis of which minimised skew to the greatest extent. The data were then Z-transformed to have a mean of zero and standard deviation of one, with extreme outliers (>3Z) discarded.

For all regression analyses a null multi-level model was run first to determine the proportion of variance explained by neighbourhood level random effects. Basic demographic information (age at assessment, sex and NS-SEC) were then added as potential *a priori* confounders with neighbourhood level predictors added subsequently using forward step-wise selection. These were only retained / added to the multivariable model if they significantly improved its fit (p<0.05; likelihood ratio test). NS-SEC and sex were coded as categorical variables, with ‘unemployed’ and ‘male’ set as reference levels.

To explore associations at the item level participants’ responses on the persecutory delusions (SAPS-D1) and (global) hallucinations (SAPS-H7) items of the SAPS

For peer review only - http://bmjopen.bmj.com/site/about/guidelines.xhtml

were also regressed on predictors. Since the distribution of participants' responses on these items could not be normalised through transformation they were re-coded as binary variables, with scores of 0-2 coded as absent-to-mild and scores of 3-5 coded as moderate-to-severe, and analyses undertaken using multi-level logistic regression.

Regression analyses were undertaken using Stata (Version 14; StataCorp LP, College Station, TX).

Patient and public involvement statement

Patients and public were not involved in the design or analysis of this study.

Results

Sample

Of the 379 participants for whom data were originally collected, 34 (8.97%) were discarded due to inappropriate residency: no fixed abode (n=5), incomplete postcode information (n=4) or resident beyond the study's catchment area (n=25). Of the remaining 345, complete symptom data were available for 335 participants (97.10%). Following transformation to minimise skew, eight outlying symptom component scores were removed. With respect to basic demographics, seven data-points were not coded at the time of data collection: age (n=2), gender (n=2), and occupation (n=3). Further, voter turn-out (SCI) was not available for three neighbourhoods due to the existence of a single, unopposed representative. Taken together, these exclusions resulted in a complete core data-set of 319 participants, representing 84.17% of the original sample (see Table 1). Within this sample the median age was 24.16 years [inter-quartile range (IQR)=20-30.23], 65.83% were male, 57.05% were unemployed and 75.86% experienced non-affective psychosis.

For peer review only - http://bmjopen.bmj.com/site/about/guidelines.xhtml

The median SAPS and SANS total global scores were 32 (IQR=23-45) and 18 (IQR=7-34), respectively.

Participants retained in the analyses did not differ from the original data-set on any of the individual or neighbourhood level variables (data available upon request).

For peer review only

For peer review only - http://bmjopen.bmj.com/site/about/guidelines.xhtml

Table 1 Demographic and clinical characteristics of the study sample. Statistics provided include the number and percentage of cases (N / %), the median and the inter-quartile range (IQR). Age=age at assessment; DUP=duration of untreated psychosis; NS-SEC= National Statistics Socio-Economic Classification system; SAPS= Scales for the Assessment of Positive symptoms (global scores); SANS= Scales for the Assessment of Negative symptoms (global scores).

Variable	Level	N (%)	Median	IQR
Age	-	-	24.16	20-30.23
Gender	*Male*	210 (65.83)	-	-
	Female	109 (34.17)	-	-
NS-SEC	*Managerial and professional*	17 (5.33)	-	-
	Intermediate occupations	21 (6.58)	-	-
	Routine and manual	50 (15.67)	-	-
	Unemployed	182 (57.05)	-	-
	Students	49 (15.36)	-	-
Diagnosis	*Non-Affective*	242 (75.86)	-	-
	Affective	71 (22.26)	-	-
	Not recorded	6 (1.88)		
SAPS total	-	-	32	23-45
SANS total	-	-	18	7-34

For peer review only

Neighbourhood data

Data included were distributed across 113 neighbourhoods from 14 boroughs. The median number of LSOAs per neighbourhood was 7 (IQR=6-8) and the median number of participants per neighbourhood was 2 (IQR=1-4). In order to characterise the neighbourhoods further the pattern of associations between key measures (Supplementary Table 1) were explored. Spearman's correlations (Supplementary Table 2) indicated that, at a corrected alpha of 0.002 (corrected for 21 comparisons), the more deprived neighbourhoods (high IMD) were significantly more densely populated ($p<0.001$), less unequal ($p<0.001$), more socially fragmented ($p<0.001$), less socially cohesive ($p<0.001$) and characterised by higher ethnic segregation ($p<0.01$) and a higher density of ethnic minorities ($p<0.001$). Further, more unequal neighbourhoods were significantly less densely populated ($p<0.001$), less deprived ($p<0.001$), less socially fragmented ($p<0.001$), more socially cohesive ($p<0.001$) and had a lower density of ethnic minorities ($p<0.001$).

Multi-level modelling of symptom components and symptom scores

Fixed effects

Analyses undertaken whilst controlling for age, gender and social class- ($\chi^2_{(7)}$=24.25, p=0.001) indicated that inequality (GINI-ID) was significantly associated with negative symptoms (Table 2 and Supplementary Figure 3): higher inequality was associated with less severe symptoms (coefficient=-1.54, CI=-2.76, -0.33, p=0.01). This effect persisted after further control for other (positive and disorganisation) symptoms (coefficient=-1.66, CI=-2.86, -0.46, $p<0.01$) as well as absolute deprivation, i.e. ID or IMD (coefficient=-2.06, CI=-3.32,-0.8, $p<0.01$; coefficient=-2.06, CI=-3.32,-0.8, $p<0.01$). Further, ethnic segregation (IDS-BME) was associated with positive symptoms after controlling for basic demographics: higher segregation of ethnic minorities was associated with less severe positive

For peer review only - http://bmjopen.bmj.com/site/about/guidelines.xhtml

symptoms (coefficient=-2.36, CI=-4.2, -0.52, p=0.01; Table 2 and Supplementary Figure 4). This effect also persisted after controlling for other symptoms (coefficient=-2.32, CI=-4.17, -0.48, p=0.01) as well as absolute deprivation, i.e. ID or IMD (coefficient=-2.2, CI=-4.09, -0.32, p=0.02; coefficient=-2.17, CI=-4.07,-0.28, p=0.03). However, no significant neighbourhood level predictors were found for disorganisation symptoms (Table 2 and Supplementary Figure 5). Nor did any neighbourhood level predictor predict significant variance in paranoid delusions or global hallucinations (symptom level analyses; Supplementary Table 6).

Random effects

Neighbourhood level random effects did not explain significant levels of variance in symptom dimension or individual symptom scores, either before or after controlling for demographic variables: negative symptoms (<0.001%), positive symptoms (<0.001%), disorganisation symptoms (1.45%), paranoid delusions (5.35%) or global hallucinations (<0.001%) (all p-values>0.05).

For peer review only - http://bmjopen.bmj.com/site/about/guidelines.xhtml

Table 2 Multi-level modelling of symptom components (summary). Individual symptom dimensions derived from second-order principal component analysis were regressed on predictor variables using multi-level regression analyses. Data reported are from multivariate models that were run whilst controlling for basic demographic information (age, gender and NS-SEC) and other symptoms (e.g. negative and disorganisation symptoms for the positive symptoms). Neighbourhood level variables are in italics. Significant variables are in bold.

		Negative symptoms		Positive symptoms		Disorganisation symptoms	
		Fixed part of the model		Fixed part of the model		Fixed part of the model	
Predictor	Level	Coefficient (95% CIs)	Wald p value	Coefficient (95% CIs)	Wald p value	Coefficient (95% CIs)	Wald p value
Age		0.01 (-0.01, 0.02)	0.37	-0.01 (-0.02, 0)	0.12	-0.01 (-0.02, 0.01)	0.33
Gender	Female	-0.36 (-0.59, -0.12)	**<0.01**	0.06 (-0.18, 0.3)	0.63	-0.23 (-0.47, 0.01)	0.06
NS-SEC	Managerial	-0.49 (-0.98, 0)	0.05	0.03 (-0.47, 0.53)	0.91	-0.22 (-0.73, 0.29)	0.39
	Intermediate	-0.23 (-0.67, 0.22)	0.32	0.49 (0.05, 0.94)	**0.03**	-0.08 (-0.54, 0.37)	0.72
	Routine	-0.35 (-0.65, -0.04)	**0.03**	0.16 (-0.15, 0.47)	0.32	-0.26 (-0.57, 0.05)	0.1
	Student	0.09 (-0.23, 0.4)	0.59	0.07 (-0.25, 0.39)	0.67	-0.21 (-0.53, 0.12)	0.21
Pop Den		-	-	-	-	-	-
ID		-	-	-	-		
IMD		-	-	-	-	-	-
GINI-ID		-1.66 (-2.86, -0.46)	**<0.01**	-	-	-	-
GINI-IMD		**-**	**-**	-	-	-	-
SFI		-	-	-	-	-	-
SCI		-	-	-	-	-	-
IDS-BME		-	-	-2.32 (-4.17, -0.48)	**0.01**	-	-
DEN-BME		-	-	-	-	-	-

For peer review only

Discussion

Fixed effects analyses revealed that each symptom dimension (positive, negative and disorganisation) was associated with a distinct pattern of neighbourhood level risk factors; thus, higher levels of inequality were associated with lower negative symptoms, and greater ethnic segregation was associated with lower positive symptoms. However, contrary to our primary hypotheses, there was no evidence to suggest that the positive symptoms of psychosis were elevated in areas scoring highly on classical indicators of environmental adversity or socioeconomic deprivation (e.g. population density, income deprivation or inequality), either at the level of the symptom cluster, or with respect to persecutory delusions or hallucinations specifically.

Nonetheless, the finding that participants living in neighbourhoods characterised by highly segregated BME communities exhibited *less* severe positive symptoms is interesting, since -perhaps counter-intuitively- a high index of dissimilarity arguably reflects a socially impoverished environment. Thus, whilst a high index of dissimilarity indicates high segregation *between* populations, it implies reduced fragmentation *within* a given ethnic group, i.e. a high potential for bonding social capital [34,35], with potential protective effects against the positive symptoms of psychosis [36]. This is consistent with a previous study undertaken in South East London, which reported a lower incidence of psychosis in areas characterised by higher (BME) ethnic segregation [6], as well as research showing that the risk of psychosis diminishes as the proportion of one's own ethnic group increases within a neighbourhood [36–39].

The finding that participants living in neighbourhoods characterised by higher inequality (GINI-ID) exhibited less severe negative symptoms is inconsistent with

For peer review only - http://bmjopen.bmj.com/site/about/guidelines.xhtml

the Income Inequality Hypothesis (IIH) [40], which posits that highly unequal neighbourhoods are characterised by poorer health outcomes. However, the empirical findings on the association between income inequality and mental health are not clear cut. A recent qualitative synthesis identified 27 relevant studies that explored this issue [41], nine of which met inclusion criteria for a meta-analysis; of these, only one found a positive association between higher inequality and poorer mental health, whilst six reported mixed results, and two found no significant effects. The authors concluded that the extant literature in the field is characterised by small effect sizes and a high level of heterogeneity in findings. See [42,43] also.

The association between inequality and psychosis -rather than mental health in general- is also not clear-cut. For example, whilst no association was seen between income inequality and psychotic *symptoms* in a study of FEP undertaken in South East London and Nottinghamshire [13], links between inequality and psychosis *incidence* or *prevalence* have been shown using sample sizes comparable to our own [1,4,44]. It is not inconceivable, however, that inequality and/or deprivation might be characterised by relatively independent (or even inverted) patterns of association with psychosis incidence and psychotic symptom severity. For example, one might imagine how in a wealthy neighbourhood, in which health and social services are well resourced (for detection as well as treatment), the recorded incidence of psychosis might be elevated but the severity of symptoms in those individuals reduced.

Another potential explanation for the unexpected finding of an association between higher inequality and lower negative symptoms scores resides in the socio-political geography of the West London region examined. According to the Social Capital Hypothesis [45] areas that are relatively more unequal, i.e. defined by a higher Gini coefficient, tend to have worse health outcomes because they are characterised by

For peer review only - http://bmjopen.bmj.com/site/about/guidelines.xhtml

lower levels of interpersonal trust and a lack of a sense of shared community (i.e. low social capital and high social fragmentation). Thus, poor social cohesion / social capital is thought to mediate the negative effects of inequality on health [46]. However, within the WLFEP data-set the inverse was true (Table 2): more unequal neighbourhoods were in fact *more* socially cohesive, characterised by extremes of affluence (rather than deprivation) and a relative abundance of social capital. Although a direct association was not seen between social cohesion and psychotic symptoms in our study, it is possible that more sensitive indices of social capital, or measures that tap into different facets of this complex construct [47], might uncover such an association [48] .

Finally, the reported association between higher inequality and lower negative symptoms might be explained by the Mixed Neighbourhood Hypothesis (MNH) [49–51]. According to the MNH, the mixing of individuals from different socioeconomic backgrounds within areas of high inequality may be protective against cultures of crime, substance use, joblessness and a lack of social opportunity that can become endemic to areas of homogenous deprivation. Further, the wealthy may also bring higher investment in local infrastructure and resources. Arguably, however, despite considerable political interest in the area, particularly in the US, reviews of the literature have typically failed to find convincing evidence for the MNH [17,41] or the benefits of mixed housing schemes that are linked to the theory [52].

With respect to the limitations of the study, its cross-sectional design precludes inferences about causality and without data on participants' ethnicity potential interactions with neighbourhood level predictors such as ethnic density and ethnic fragmentation could not be explored. Finally, since our sample was characterised by relatively few participants per neighbourhood we were likely under-powered to detect random effects, particularly in view of the fact that estimates of random

For peer review only - http://bmjopen.bmj.com/site/about/guidelines.xhtml

effects are comparatively unreliable and biased towards under-estimation [53]. Nonetheless, consistent with our *a priori* aims, neighbourhood covariates (i.e. fixed effects) were still modelled and significant associations found.

In conclusion, the findings reported contribute to a growing body of evidence that highlights the importance of social and socioeconomic factors in the expression of psychosis symptoms. Further, they suggest that associations between psychosis and the environment may be operating at the level of symptoms (rather than at the level of incidence / prevalence), with specific environmental predictors linked to specific symptoms or symptoms clusters. Arguably, this lends support to current calls in both clinical [54] and research [55] practice for a shift away from categorical / diagnostic systems of classification towards dimension based models of psychosis [56–58], a debate that is also echoed more broadly in the field of general psychopathology [59,60]. Finally, the findings also highlight the need for longitudinal research that can begin to elucidate the underlying mechanisms that link the environment to symptom expression, in addition to establishing the direction of causality.

Author contributions: EJ and TB designed and oversaw the original West London First Episode Psychosis Study (WLFEP). SM and IH conducted clinical interviews with participants. VH and MT designed the current study. MT undertook all analyses and wrote the first draft of the manuscript. JK provided advice and support on statistical analyses. All authors commented on -and contributed to- the final version of the manuscript.

For peer review only - http://bmjopen.bmj.com/site/about/guidelines.xhtml

References

1 Kirkbride JB, Jones PB, Ullrich S, *et al.* Social deprivation, inequality, and the neighborhood-level incidence of psychotic syndromes in East London. *Schizophr Bull* 2014;**40**:169–80.http://ovidsp.ovid.com/ovidweb.cgi?T=JS&PAGE=reference&D=psyc11&NEWS=N&AN=2014-05521-022

2 Heinz A, Deserno L, Reininghaus U. Urbanicity, social adversity and psychosis. *World Psychiatry* 2013;**12**:187–97. doi:10.1002/wps.20056

3 O'Donoghue B, Roche E, Lane A. Neighbourhood level social deprivation and the risk of psychotic disorders: a systematic review. *Soc Psychiatry Psychiatr Epidemiol* 2016;**51**:941–50. doi:10.1007/s00127-016-1233-4

4 Burns JK, Esterhuizen T. Poverty, inequality and the treated incidence of first-episode psychosis: an ecological study from South Africa. *Soc Psychiatry Psychiatr Epidemiol* 2008;**43**:331–5. doi:10.1007/s00127-008-0308-2

5 Burns JK, Tomita A, Kapadia AS. Income inequality and schizophrenia: increased schizophrenia incidence in countries with high levels of income inequality. *Int J Soc Psychiatry* 2014;**60**:185–96. doi:10.1177/0020764013481426

6 Kirkbride JB, Morgan C, Fearon P, *et al.* Neighbourhood-level effects on psychoses: re-examining the role of context. *Psychol Med* 2007;**37**:1413–25. doi:10.1017/S0033291707000499

7 Bentall RP, de Sousa P, Varese F, *et al.* From adversity to psychosis: pathways and mechanisms from specific adversities to specific symptoms. *Soc Psychiatry Psychiatr Epidemiol* 2014;**49**:1011–22. doi:10.1007/s00127-014-0914-0

8 Ellett L, Freeman D, Garety P. The psychological effect of an urban environment on individuals with persecutory delusions: the Camberwell walk

For peer review only - http://bmjopen.bmj.com/site/about/guidelines.xhtml

study. *Schizophr Res* 2008;**99**:77–84. doi:10.1016/j.schres.2007.10.027

9 Veling W, Pot-Kolder R, Counotte J, *et al.* Environmental Social Stress, Paranoia and Psychosis Liability: A Virtual Reality Study. *Schizophr Bull* 2016;**42**:1363–71. doi:10.1093/schbul/sbw031

10 Bentall RP, Corcoran R, Howard R, *et al.* Persecutory delusions: a review and theoretical integration. *Clin Psychol Rev* 2001;**21**:1143–92.http://www.ncbi.nlm.nih.gov/pubmed/11702511 (accessed 14 Nov 2018).

11 Freeman D, Garety PA, Kuipers E, *et al.* A cognitive model of persecutory delusions. *Br J Clin Psychol* 2002;**41**:331–47.http://www.ncbi.nlm.nih.gov/pubmed/12437789 (accessed 14 Nov 2018).

12 Mirowsky J, Ross CE. Paranoia and the structure of powerlessness. *Am Sociol Rev* 1983;**48**:228–39.http://www.ncbi.nlm.nih.gov/pubmed/6859680 (accessed 14 Nov 2018).

13 Oher FJ, Demjaha A, Jackson D, *et al.* The effect of the environment on symptom dimensions in the first episode of psychosis: a multilevel study. *Psychol Med* 2014;**44**:2419–30. doi:10.1017/S0033291713003188

14 Wickham S, Taylor P, Shevlin M, *et al.* The Impact of Social Deprivation on Paranoia, Hallucinations, Mania and Depression: The Role of Discrimination Social Support, Stress and Trust. *PLoS One* 2014;**9**. doi:10.1371/journal.pone.0105140

15 Johnson SL, Wibbels E, Wilkinson R. Economic inequality is related to cross-national prevalence of psychotic symptoms. Soc. Psychiatry Psychiatr. Epidemiol. 2015;:No-Specified.http://ovidsp.ovid.com/ovidweb.cgi?T=JS&PAGE=reference&D=psyc11&NEWS=N&AN=2015-42803-001

16 Newbury J, Arseneault L, Caspi A, *et al.* Why Are Children in Urban Neighborhoods at Increased Risk for Psychotic Symptoms? Findings From a UK Longitudinal Cohort Study. *Schizophr Bull* 2016;**42**:1372–83.

For peer review only

For peer review only - http://bmjopen.bmj.com/site/about/guidelines.xhtml

doi:10.1093/schbul/sbw052

17 Tibber MS, Kirkbride JB, Joyce EM, *et al.* The component structure of the scales for the assessment of positive and negative symptoms in first-episode psychosis and its dependence on variations in analytic methods. *Psychiatry Res* 2018;**270**:869–79. doi:10.1016/J.PSYCHRES.2018.10.046

18 Johnson SL, Wibbels E, Wilkinson R. Economic inequality is related to cross-national prevalence of psychotic symptoms. *Soc Psychiatry Psychiatr Epidemiol* 2015;**50**:1799–807.http://ovidsp.ovid.com/ovidweb.cgi?T=JS&PAGE=reference&D=medl&NEWS=N&AN=26370212

19 Barnes TR, Hutton SB, Chapman MJ, *et al.* West London first-episode study of schizophrenia. Clinical correlates of duration of untreated psychosis. *Br J Psychiatry* 2000;**177**:207–11.http://www.ncbi.nlm.nih.gov/pubmed/11040879 (accessed 6 Mar 2017).

20 Joyce EM, Hutton SB, Mutsatsa SH, *et al.* Cognitive heterogeneity in first-episode schizophrenia. *Br J Psychiatry* 2005;**187**:516–22. doi:10.1192/bjp.187.6.516

21 Jablensky A, Sartorius N, Ernberg G, *et al.* Schizophrenia: manifestations, incidence and course in different cultures. A World Health Organization ten-country study. *Psychol Med Monogr Suppl* 1992;**20**:1–97.http://www.ncbi.nlm.nih.gov/pubmed/1565705 (accessed 29 May 2017).

22 American Psychiatric Association. *Diagnostic and Statistical Manual of Mental Disorders - Third Edition*. III. Washington, DC: : American Psychiatric Association 1980.

23 American Psychiatric Association. *Diagnostic and Statistical Manual of Mental Disorders - Fourth Edition*. 4th ed. Washington, DC: : American Psychiatric Association 2000.

24 Castle DJ, Jablensky A, McGrath JJ, *et al.* The diagnostic interview for

For peer review only - http://bmjopen.bmj.com/site/about/guidelines.xhtml

For peer review only

psychoses (DIP): development, reliability and applications. *Psychol Med* 2006;**36**:69–80. doi:10.1017/S0033291705005969

25 Huddy VC, Hodgson TL, Kapasi M, *et al.* Gaze strategies during planning in first-episode psychosis. *J Abnorm Psychol* 2007;**116**:589–98. doi:10.1037/0021-843X.116.3.589

26 Andreasen N. Methods for assessing positive and negative symptoms. In: Andreasen N, ed. *Schizophrenia : Positive and Negative Symptoms and Syndromes. Modern Problems in Pharmacopsychiatry*. Basel: : Karger 1990. 73–85.

27 Peralta V, Cuesta MJ. Dimensional structure of psychotic symptoms: an item-level analysis of SAPS and SANS symptoms in psychotic disorders. *Schizophr Res* 1999;**38**:13–26.http://www.ncbi.nlm.nih.gov/pubmed/10427607 (accessed 26 Jul 2015).

28 Rose D, Pevalin D. The National Statistics Socio-economic Classification: Origins, Development and Use. Norwich: 2005.

29 Office for National Statistics. Nomis - official labour market statistics - 2001 census data. https://www.nomisweb.co.uk

30 Kaymaz N, Krabbendam L, de Graaf R, *et al.* Evidence that the urban environment specifically impacts on the psychotic but not the affective dimension of bipolar disorder. *Soc Psychiatry Psychiatr Epidemiol* 2006;**41**:679–85. doi:10.1007/s00127-006-0086-7

31 Congdon P. Suicide and Parasuicide in London: A Small-area Study. *Urban Stud* 1996;**33**:137–58. doi:10.1080/00420989650012194

32 Allardyce J, Gilmour H, Atkinson JM, *et al.* Social fragmentation, deprivation and urbanicity: relation to first-admission rates for psychoses. *Br J Psychiatry* Published Online First: 1 November 2005. doi:10.1192/bjp.187.5.401 <http://dx.doi.org/10.1192/bjp.187.5.401>)

33 Rallings C, Thrasher M. Local Elections Handbook 2002. Plymouth: 2002.

For peer review only - http://bmjopen.bmj.com/site/about/guidelines.xhtml

http://www.electionscentre.co.uk/?page_id=3684

34 Becares L, Nazroo J. Social capital, ethnic density and mental health among ethnic minority people in England: a mixed-methods study. *Ethn Health* 2013;**18**:544–62. doi:10.1080/13557858.2013.828831

35 Orford J. Two Concepts for Understanding Inequalities by Position and Place - social capital and sense of community. In: *Community Psychology - Challenges, Controversies and Emerging Consensus*. Chichester: : John Wiley & Sons Ltd. 2008. 163–81.

36 Das-Munshi J, Bécares L, Boydell JE, *et al.* Ethnic density as a buffer for psychotic experiences: findings from a national survey (EMPIRIC). *Br J Psychiatry* 2012;**201**.http://bjp.rcpsych.org/content/201/4/282 (accessed 19 May 2017).

37 Boydell J, van Os J, McKenzie K, *et al.* Incidence of schizophrenia in ethnic minorities in London: ecological study into interactions with environment. *BMJ* 2001;**323**:1336–8.http://www.ncbi.nlm.nih.gov/pubmed/11739218 (accessed 20 Mar 2017).

38 Schofield P, Thygesen M, Das-Munshi J, *et al.* Ethnic density, urbanicity and psychosis risk for migrant groups – A population cohort study. *Schizophr Res* 2017;**190**:82–7. doi:10.1016/j.schres.2017.03.032

39 Veling W, Susser E, van Os J, *et al.* Ethnic Density of Neighborhoods and Incidence of Psychotic Disorders Among Immigrants. *Am J Psychiatry* 2008;**165**:66–73. doi:10.1176/appi.ajp.2007.07030423

40 Wilkinson RG, Pickett K. *The Spirit Level*. London: : The Penguin Group 2009.

41 Ribeiro WS, Bauer A, Andrade MCR, *et al.* Income inequality and mental illness-related morbidity and resilience: a systematic review and meta-analysis. *The Lancet Psychiatry* 2017;**4**:554–62. doi:10.1016/S2215-0366(17)30159-1

For peer review only

42 Wilkinson R, Pickett K. *The Inner Level - How More Equal Societies Reduce Stress, Restore Sanity and Improve Everyone's Wellbeing*. First. St Ives: : Penguin Random House 2018.

43 Tibber M. *Individual- and neighbourhood-level predictors of psychotic symptom dimensions in West London.* 2017.http://discovery.ucl.ac.uk/1575465/

44 Boydell J, van Os J, McKenzie K, *et al.* The association of inequality with the incidence of schizophrenia - An ecological study. *Soc Psychiatry Psychiatr Epidemiol* 2004;**39**:597–9. doi:10.1007/s00127-004-0789-6

45 McKenzie K, Whitely R, Weich S. Social capital and mental health. *Br J Psychiatry* 2002;**181**.

46 Kragten N, Rözer J. The Income Inequality Hypothesis Revisited: Assessing the Hypothesis Using Four Methodological Approaches. *Soc Indic Res* 2017;**131**:1015–33. doi:10.1007/s11205-016-1283-8

47 Siegler V. Measuring Social Capital. 2014.

48 Clough-Gorr KM, Egger M, Spoerri A. A Swiss paradox? Higher income inequality of municipalities is associated with lower mortality in Switzerland. *Eur J Epidemiol* 2015;**30**:627–36. doi:10.1007/s10654-015-9987-7

49 Manley D, VanHam M, Doherty J. Social mixing as a cure for negative neighbourhood effects: evidence based policy or urban myth? In: Bridge G, Butler T, Lees L, eds. *Mixed Communities: gentrification by stealth?* Bristol: : The Policy Press 2012. 1–17.

50 Musterd S, Andersson R. Housing Mix, Social Mix, and Social Opportunities. *Urban Aff Rev* 2005;**40**:761–90. doi:10.1177/1078087405276006

51 Ostendorf W, Musterd S, De Vos S. Social Mix and the Neighbourhood Effect. Policy Ambitions and Empirical Evidence. *Hous Stud* 2001;**16**:371–80. doi:10.1080/02673030120049724

52 Melis G, Marra G, Gelormino E. Housing and Social Mix - Equity Action.

For peer review only - http://bmjopen.bmj.com/site/about/guidelines.xhtml

2013. http://www.health-inequalities.eu/wp-content/uploads/2016/05/Literature-Review-housing_social-mix-final.pdf

53 Bryan ML, Jenkins SP. Multilevel Modelling of Country Effects: A Cautionary Tale. *Eur Sociol Rev* 2016;**32**:3–22. doi:10.1093/esr/jcv059

54 Parker GF. DSM-5 and Psychotic and Mood Disorders. *J Am Acad Psychiatry Law Online* 2014;**42**.http://jaapl.org/content/42/2/182 (accessed 19 May 2017).

55 Morris SE, Cuthbert BN. Research Domain Criteria: cognitive systems, neural circuits, and dimensions of behavior. *Dialogues Clin Neurosci* 2012;**14**:29–37.http://www.ncbi.nlm.nih.gov/pubmed/22577302 (accessed 16 May 2017).

56 Allardyce J, McCreadie RG, Morrison G, *et al.* Do symptom dimensions or categorical diagnoses best discriminate between known risk factors for psychosis? *Soc Psychiatry Psychiatr Epidemiol* 2007;**42**:429–37. doi:10.1007/s00127-007-0179-y

57 Van Os J, Gilvarry C, Bale R, *et al.* A comparison of the utility of dimensional and categorical representations of psychosis. UK700 Group. *Psychol Med* 1999;**29**:595–606.http://www.ncbi.nlm.nih.gov/pubmed/10405080 (accessed 11 Aug 2015).

58 Van Os J. The transdiagnostic dimension of psychosis: implications for psychiatric nosology and research. *Shanghai Arch psychiatry* 2015;**27**:82–6. doi:10.11919/j.issn.1002-0829.215041

59 Lahey BB, Zald DH, Perkins SF, *et al.* Measuring the hierarchical general factor model of psychopathology in young adults. *Int J Methods Psychiatr Res* 2018;**27**:e1593. doi:10.1002/mpr.1593

60 Kotov R, Krueger RF, Watson D. A paradigm shift in psychiatric classification: the Hierarchical Taxonomy Of Psychopathology (HiTOP). *World Psychiatry* 2018;**17**:24–5. doi:10.1002/wps.20478

For peer review only - http://bmjopen.bmj.com/site/about/guidelines.xhtml

For peer review only

For peer review only - http://bmjopen.bmj.com/site/about/guidelines.xhtml

Supplementary Table 1 Key statistics for neighbourhood level variables (N=113 unique neighbourhoods included in the analysis). Statistics provided include the number of entries (N), the median and the interquartile range (IQR). The same data are also shown for all neighbourhoods in England (N=7932) for comparison, or else, all neighbourhoods with more than one composite LSOA (N=6685) where this was required to calculate an index (e.g. GINI-ID). Population density=general population density in people per hectare; ID=income deprivation; IMD=index of multiple deprivation; GINI-ID=Gini coefficient based on income deprivation; GINI-IMD=Gini coefficient based on index of multiple deprivation; SFI=social fragmentation index; SCI=social cohesion index; IDS-BME=index of dissimilarity for BME versus white populations; DEN-BME=BME ethnic density (people per hectare).

	Study data		National data	
	N	Median (IQR)	N	Median (IQR)
Pop density	113	63.91 (40.45-86.68)	7932	12.01 (0.02-236.05)
ID	113	11 (7.09-18)	7932	8.66 (1.03-61.71)
IMD	113	16.65 (10.2-24.33)	7932	14.68 (1.1-79.81)
GINI-ID	113	0.24 (0.18-0.3)	6685	0.21 (0-0.57)
GINI-IMD	113	0.17 (0.12-0.21)	6685	0.17 (0-0.65)
SFI	113	5.1 (1.76-7.69)	7932	-0.83 (-6.7-22.48)
SCI	113	33 (28.45-38.05)	7932	33.3
IDS-BME	113	0.13 (0.11-0.19)	6685	0.21 (0-0.79)
DEN-BME	113	0.17 (0.1-0.28)	7932	0.01 (0-0.86)

Supplementary Table 2 Spearman's correlations between neighbourhood level variables (n=113 unique neighbourhoods). Provided statistics include Spearman's Rho and p value (in brackets). Significant correlations at an alpha of 0.002 (corrected for 21 comparisons) are shown in bold. POP DENS=population density; IMD=index of multiple deprivation; GINI-IMD=Gini coefficient calculated for the index of multiple deprivation; SFI=social fragmentation index; SCI=social cohesion index (voter turnout); IDS-BME=index of dissimilarity for BME versus white populations; DEN-BME=BME ethnic density.

	IMD	GINI -IMD	SFI	SCI	IDS-BME	DEN-BME
POP DENS	**0.38 (<0.001)**	**-0.31 (<0.001)**	**0.65 (<0.001)**	**-0.42 (<0.001)**	0.24 (<0.01)	**0.31 (<0.001)**
IMD		**-0.38 (<0.001)**	**0.32 (<0.001)**	**-0.62 (<0.001)**	**0.29 (<0.01)**	**0.75 (<0.001)**
GINI-IMD		-	**-0.32 (<0.001)**	**0.45 (<0.001)**	0.05 (0.59)	**-0.31 (<0.001)**
SFI			-	**-0.41 (<0.001)**	0.09 (0.33)	0.05 (0.6)
SCI				-	-0.18 (0.06)	**-0.59 (<0.001)**
IDS-BME					-	**0.3 (<0.01)**

For peer review only

Supplementary Table 3 Negative symptoms, derived from second- order principle components analysis of individual item scores, regressed on predictor variables. The top line of the table indicates a random effects only model. Columns three and four show the results of a series of univariate analyses, examining the effects of adding individual predictors to a model that controls for core demographics only (age, gender and socioeconomic status). Model one shows the results of a multivariate analysis that includes all variables selected for inclusion. Model two is identical to model one except that other symptoms (positive and disorganisation symptoms) are also controlled for. Neighbourhood level variables are in italics. Significant p values are in bold.

		Univariate (controlling for basic demographics only)					Model 1 – Multivariate (basic)		Model 2 – Multivariate (controlling for other Sxs also)	
		Fixed part of model		Random part of model			Fixed part of the model		Fixed part of the model	
Predictor	Level	Coefficient (95% CIs)	Wald p value	Neighbourhood level variance	Chi-squared (1 df)	Chi-squared p value	Coefficient (95% CIs)	Wald p value	Coefficient (95% CIs)	Wald p value
Random only		-	-	0	0	1	-	-	-	-
Age		0.01 (-0.01, 0.02)	0.45	0	0	1	0.01 (-0.01, 0.02)	0.33	0.01 (-0.01, 0.02)	0.37
Gender	Female	-0.32 (-0.56, -0.08)	**<0.01**	0	0	1	-0.33 (-0.56, -0.09)	**<0.01**	-0.36 (-0.59, -0.12)	**<0.01**
NS-SEC	Managerial	-0.51 (-1.01, -0.01)	0.05	0	0	1	-0.47 (-0.97, 0.03)	0.06	-0.49 (-0.98, 0)	0.05
	Intermediate	-0.26 (-0.71, 0.19)	0.26	0	0	1	-0.2 (-0.65, 0.24)	0.37	-0.23 (-0.67, 0.22)	0.32
	Routine	-0.33 (-0.64, -0.02)	**0.03**	0	0	1	-0.31 (-0.61, -0.01)	**<0.05**	-0.35 (-0.65, -0.04)	**0.03**
	Student	0.08 (-0.24, 0.41)	0.61	0	0	1	0.12 (-0.2, 0.44)	0.46	0.09 (-0.23, 0.4)	0.59
Pop Den		0 (0, 0)	0.35	0	0	1	-	-	-	-
ID		-0.01 (-0.02, 0.01)	0.25	0	0	1	-	-	-	-
IMD		-0.01 (-0.02, 0.01)	0.39	0	0	1	-	-	-	-
GINI-ID		-1.54 (-2.76, -0.33)	**0.01**	0	0	1	-1.54 (-2.76, -0.33)	**0.01**	-1.66 (-2.86, -0.46)	**<0.01**
GINI-IMD		-1.42 (-2.93, 0.09)	0.07	0	0	1	-	-	**-**	**-**
SFI		-0.02 (-0.05, 0.01)	0.24	0	0	1	-	-	-	-
SCI		0 (-0.02, 0.02)	0.77	0	0	1	-	-	-	-
IDS-BME		-1.51 (-3.35, 0.33)	0.11	0	0	1	-	-	-	-
DEN-BME		0.33 (-0.27, 0.93)	0.28	0	0	1	-	-	-	-

For peer review only

Supplementary Table 4 Positive symptoms, derived from second- order principle components analysis of individual item scores, regressed on predictor variables. The top line of the table indicates a random effects only model. Columns three and four show the results of a series of univariate analyses, examining the effects of adding individual predictors to a model that controls for core demographics only (age, gender and socioeconomic status). Model one shows the results of a multivariate analysis that includes all variables selected for inclusion. Model two is identical to model one except that other symptoms (negative and disorganisation symptoms) are also controlled for. Neighbourhood level variables are in italics. Significant p values are in bold.

		Univariate (controlling for basic demographics only)					Model 1 –Multivariate (basic)		Model 2 –Multivariate (controlling for other Sxs also)	
		Fixed part of model		Random part of model			Fixed part of the model		Fixed part of the model	
Predictor	Level	Coefficient (95% CIs)	Wald p value	Neighbourhood level variance	Chi-squared (1 df)	Chi-squared p value	Coefficient (95% CIs)	Wald p value	Coefficient (95% CIs)	Wald p value
Random only		-	-	0	0	1	-	-	-	-
Age		-0.01 (-0.02, 0)	0.11	0	0	1	-0.01 (-0.02, 0)	0.12	-0.01 (-0.02, 0)	0.12
Gender	Female	0.04 (-0.2, 0.28)	0.75	0	0	1	0.05 (-0.19, 0.28)	0.7	0.06 (-0.18, 0.3)	0.63
NS-SEC	Managerial	-0.04 (-0.55, 0.46)	0.86	0	0	1	0.01 (-0.49, 0.51)	0.96	0.03 (-0.47, 0.53)	0.91
	Intermediate	0.47 (0.01, 0.92)	**0.04**	0	0	1	0.49 (0.04, 0.93)	**0.03**	0.49 (0.05, 0.94)	**0.03**
	Routine	0.15 (-0.16, 0.46)	0.35	0	0	1	0.14 (-0.16, 0.45)	0.35	0.16 (-0.15, 0.47)	0.32
	Student	0.11 (-0.22, 0.43)	0.51	0	0	1	0.07 (-0.25, 0.39)	0.68	0.07 (-0.25, 0.39)	0.67
Pop Den		0 (0, 0)	0.49	0	0	1	-	-	-	-
ID		-0.01 (-0.02, 0.01)	0.27	0	0	1	-	-	-	-
IMD		-0.01 (-0.02, 0)	0.22	0	0	1	-	-	-	-
GINI-ID		0.42 (-0.81, 1.65)	0.5	0	0	1	-	-	-	-
GINI-IMD		0.58 (-0.94, 2.1)	0.46	0	0	1	-	-	-	-
SFI		0.01 (-0.02, 0.04)	0.72	0	0	1	-	-	-	-
SCI		0.02 (0, 0.04)	0.08	0	0	1	-	-	-	-
IDS-BME		-2.36 (-4.2, -0.52)	**0.01**	0	0	1	-2.36 (-4.2, -0.52)	**0.01**	-2.32 (-4.17, -0.48)	**0.01**
DEN-BME		-0.55 (-1.15, 0.05)	0.07	0	0	1	-	-	-	-

For peer review only

Supplementary Table 5 Disorganisation symptoms, derived from second- order principle components analysis of individual item scores, regressed on predictor variables. The top line of the table indicates a random effects only model. Columns three and four show the results of a series of univariate analyses, examining the effects of adding individual predictors to a model that controls for core demographics only (age, gender and socioeconomic status). Model one shows the results of a multivariate analysis that includes all variables selected for inclusion. Model two is identical to model one except that other symptoms (negative and positive symptoms) are also controlled for. Neighbourhood level variables are in italics. Significant p values are in bold.

		Univariate (controlling for basic demographics only)					Model 1 –Multivariate (basic)		Model 2 –Multivariate (controlling for other Sxs also)	
		Fixed part of model		Random part of model			Fixed part of the model		Fixed part of the model	
Predictor	Level	Coefficient (95% CIs)	Wald p value	Neighbourhood level variance	Chi-squared (1 df)	Chi-squared p value	Coefficient (95% CIs)	Wald p value	Coefficient (95% CIs)	Wald p value
Random only		-	-	0.45	0.01	0.46	-	-	-	-
Age		-0.01 (-0.02, 0.01)	0.27	1.45	0.01	0.38	-0.01 (-0.02, 0.01)	0.27	-0.01 (-0.02, 0.01)	0.33
Gender	*Female*	-0.18 (-0.42, 0.06)	0.14	1.45	0.01	0.38	-0.18 (-0.42, 0.06)	0.14	-0.23 (-0.47, 0.01)	0.06
NS-SEC	*Managerial*	-0.15 (-0.65, 0.36)	0.57	1.45	0.01	0.38	-0.15 (-0.65, 0.36)	0.57	-0.22 (-0.73, 0.29)	0.39
	Intermediate	-0.04 (-0.5, 0.42)	0.86	1.45	0.01	0.38	-0.04 (-0.5, 0.42)	0.86	-0.08 (-0.54, 0.37)	0.72
	Routine	-0.21 (-0.52, 0.1)	0.19	1.45	0.01	0.38	-0.21 (-0.52, 0.1)	0.19	-0.26 (-0.57, 0.05)	0.1
	Student	-0.22 (-0.54, 0.11)	0.19	1.45	0.01	0.38	-0.22 (-0.54, 0.11)	0.19	-0.21 (-0.53, 0.12)	0.21
Pop Den		0 (0, 0)	0.53	1.1	0.05	0.41	-	-	-	-
ID		0.01 (-0.01, 0.02)	0.31	1.28	0.07	0.39	-	-		
IMD		0.01 (0, 0.02)	0.25	1.1	0.05	0.41	-	-	-	-
GINI-ID		-0.65 (-1.9, 0.6)	0.23	0.85	0.03	0.43	-	-	-	-
GINI-IMD		-0.84 (-2.39, 0.71)	0.29	0.86	0.03	0.43	-	-	-	-
SFI		0 (-0.03, 0.04)	0.76	1.41	0.09	0.38	-	-	-	-
SCI		-0.01 (-0.03, 0)	0.14	0.76	0.03	0.44	-	-	-	-
IDS-BME		0.29 (-1.62, 2.19)	0.77	1.37	0.08	0.39	-	-	-	-
DEN-BME		0.23 (-0.38, 0.85)	0.46	0.87	0.03	0.43	-	-	-	-

For peer review only

For peer review only - http://bmjopen.bmj.com/site/about/guidelines.xhtml

Supplementary Table 6 Multi-level modelling of paranoid delusions and global hallucinations. Relevant scores were regressed on predictor variables using multi-level logistic analyses. Significant p values are in bold.

		Persecutory delusions		Global hallucinations	
		Fixed part of the model		Fixed part of the model	
Predictor	Level	Odds ratio (95% CIs)	Wald p value	Odds ratio (95% CIs)	Wald p value
Age		1.03 (1, 1.06)	0.09	0.99 (0.96, 1.02)	0.36
Gender	Female	1.01 (0.59, 1.72)	0.97	0.86 (0.52, 1.44)	0.57
NS-SEC	Managerial	0.22 (0.07, 0.66)	**<0.01**	1.58 (0.51, 4.9)	0.43
	Intermediate	0.91 (0.32, 2.59)	0.86	1 (0.38, 2.59)	1
	Routine	0.75 (0.38, 1.46)	0.39	1.04 (0.55, 2)	0.9
	Student	0.72 (0.36, 1.44)	0.36	2.11 (0.97, 4.63)	0.06

For peer review only

BMJ Open

Are socio-environmental factors associated with psychotic symptoms in people with first-episode psychosis?: a cross-sectional study of a west London clinical sample

Journal:	*BMJ Open*
Manuscript ID	bmjopen-2019-030448.R1
Article Type:	Research
Date Submitted by the Author:	06-Aug-2019
Complete List of Authors:	Tibber, Marc; University College London, Department of Clinical, Educational and Health Psychology;<br>Kirkbride, James; University College London, Division of Psychiatry<br>Mutsatsa, Stanley ; School of Health Sciences, City, University of London<br>Harrison, Isobel ; University College London, Division of Psychiatry<br>Barnes, Thomas; Imperial College London, Centre for Psychiatry<br>Joyce, Eileen; University College London, Institute of Neurology<br>Huddy, Vyv; University of Sheffield, Clinical Psychology Unit, Department of Psychology
**Primary Subject Heading**:	Epidemiology
Secondary Subject Heading:	Mental health
Keywords:	Schizophrenia & psychotic disorders < PSYCHIATRY, EPIDEMIOLOGY, Adult psychiatry < PSYCHIATRY, Inequality, Deprivation, Social capital

SCHOLARONE™
Manuscripts

Are socio-environmental factors associated with psychotic symptoms in people with first-episode psychosis?: a cross-sectional study of a west London clinical sample

Marc S. Tibber[a], James B. Kirkbride[b], Stanley Mutsatsa[c], Isobel Harrison[b], Thomas R. E. Barnes[d], Eileen M. Joyce[e] ,Vyv Huddy[f]

[a]Department of Clinical, Educational and Health Psychology, UCL, London, UK; [b]Division of Psychiatry, UCL, London, UK; [c]School of Health Sciences, City, University of London, UK; [d]Centre for Psychiatry, Imperial College London, UK; [e]UCL Institute of Neurology, London, UK; [f]Clinical Psychology Unit, Department of Psychology, University of Sheffield, UK.

Corresponding author:
Marc Tibber
Email: m.tibber@ucl.ac.uk
0 44 (0)20 7679 1673.

For peer review only

For peer review only - http://bmjopen.bmj.com/site/about/guidelines.xhtml

Abstract

Objectives: To determine whether neighbourhood level socio-environmental factors including deprivation and inequality predict variance in psychotic symptoms after controlling for individual level demographics.

Design: A cross-sectional design was employed.

Setting: Data were originally collected from secondary care services within the UK boroughs of Ealing, Hammersmith and Fulham, Wandsworth, Kingston, Richmond, Merton, Sutton and Hounslow as part of the West London First-Episode Psychosis study.

Participants: Complete case analyses were undertaken on 319 participants who met the following inclusion criteria: aged 16 years or over, resident in the study's catchment area, experiencing a first psychotic episode, with fewer than 12 weeks exposure to antipsychotic medication and sufficient command of English to facilitate assessment.

Outcome measures: Symptom dimension scores, derived from principal components analyses of the SAPS and SANS, were regressed on neighbourhood level predictors, including population density, income deprivation, income inequality, social fragmentation, social cohesion, ethnic density and ethnic fragmentation, using multi-level regression. Whilst age, gender and socioeconomic status were included as individual level covariates, data on participant ethnicity were not available.

Results: Higher income inequality was associated with lower negative symptom scores (coefficient=-1.66, CI=-2.86, -0.46, p<0.01) and higher levels of ethnic segregation were associated with lower positive symptom scores (coefficient=-2.32, CI=-4.17, -0.48, p=0.01) after adjustment for covariates.

Conclusions: These findings provide further evidence that particular characteristics of the environment may be linked to specific symptom clusters in psychosis.

For peer review only - http://bmjopen.bmj.com/site/about/guidelines.xhtml

Longitudinal studies are required to begin to tease apart the underlying mechanisms involved as well as the causal direction of such associations.

Strengths and limitations of the study:

- This study goes beyond incidence / prevalence level research to explore the environmental predictors of psychosis at the resolution of symptoms and symptom clusters.
- This symptom level approach has the potential to further our understanding of underlying etiological mechanisms and is in line with current developments in clinical and research practice.
- The study uses a multi-level analysis approach that is perfectly suited to the nested / hierarchical structure of the data.
- The main limitation of the study is its cross-sectional design, which precludes inferences about causality.

Funding: The West London Study of First Episode Psychosis was funded by a Wellcome Trust Programme grant to Eileen Joyce, Thomas Barnes, Maria Ron and Gareth Barker (grant number 064607/Z/01/Z). James Kirkbride was supported by a Sir Henry Dale Fellowship, jointly funded by the Wellcome Trust and the Royal Society (Grant no. 101272/Z/13/Z). Eileen Joyce and James Kirkbride were supported by the National Institute for Health Research University College London Hospitals Biomedical Research Centre. On behalf of all authors, the corresponding author states that there is no conflict of interest.

Competing interests: None to declare.

For peer review only - http://bmjopen.bmj.com/site/about/guidelines.xhtml

Background

Epidemiological studies exploring geographical variation in the incidence of psychotic disorders have highlighted various characteristics of environments that are typically associated with an increased risk including high population density [1], urbanicity [2], income deprivation [3], income inequality [1,4,5] and low social cohesion [6]. More recently, researchers have begun to investigate whether such risk factors are associated not with the *incidence* of psychosis, but with the *severity* of psychosis symptoms or symptom cluster scores. Underlying this approach is the notion that exposure to particular types of adverse environments and experiences may be causally linked to the development or exacerbation of specific psychological symptoms [7]. For example, it has been suggested that densely-populated, deprived, inner-city areas may be causally associated with the development and expression of positive symptoms, e.g. paranoid delusions and hallucinations [8,9]. It has been hypothesised that this association may be mediated, at least in part, by the increased sense of disempowerment, victimisation and social alienation that (arguably) characterise such neighbourhoods [10–12].

To date only a handful of studies have used an epidemiological approach to explore the association between socio-environmental adversity and specific psychosis symptoms or symptom clusters. Whilst these studies have typically reported associations between indices of environmental adversity and some form of positive symptom expression, associations with specific symptom items were not consistent across studies. For example, whilst Oher et al. [13] reported a significant association between neighbourhood level population density and hallucinations but *not* paranoia in first episode psychosis (FEP), Wickham et al. [14] found an inverse pattern of associations with neighbourhood level deprivation in the general population: the most deprived neighbourhoods were associated with increased paranoia but *not* hallucinations. In addition, reported associations between socio-environmental

For peer review only - http://bmjopen.bmj.com/site/about/guidelines.xhtml

adversity and symptom severity in these studies were not restricted to positive symptoms; associations were also seen with depressive symptoms [13,14]. (See [15,16] also).

It is unclear whether this variation -or arguably discrepancy- in findings is due to small effect sizes / chance, or instead, methodological differences between studies. For example, the studies described were undertaken on different population samples (clinical vs. non-clinical) at different levels of analysis (within a country at the level of neighbourhoods vs. across countries) using distinct indices of socio-environmental adversity (urbanicity, deprivation and/or inequality) and different symptom measures [13,14]. Nonetheless, the possibility that specific symptom clusters may be linked to defined socio-environmental risk factors suggests value in moving away from studies of diagnostic incidence towards a focus on the predictors of psychotic symptoms and symptom clusters. By virtue of their greater specificity symptom level analyses of this kind may be more informative as to the underlying aetiological mechanisms involved, as well as potential targets for intervention.

In a recent study of the symptom dimensions underlying psychotic disorders in a first episode psychosis (FEP) cohort in West London we have shown that symptoms were best characterised by positive, negative and disorganisation syndromes [17]. Here, we examined the extent to which these three symptom dimensions were predicted by neighbourhood level socio-environmental factors, including indices of deprivation, inequality and social capital. Given the extant literature in the field [13,14,16,18] we predicted that at the syndrome level, indices of socio-environmental adversity would be associated with higher levels of positive symptoms. Further, given the findings reported by Oher et al. [13] in their FEP sample (which most closely matches our own), at an item level of analysis we

For peer review only - http://bmjopen.bmj.com/site/about/guidelines.xhtml

predicted that socio-environmental adversity would predict hallucinations, but not paranoia.

Methods

Setting

Cross-sectional data were originally collected as part of the prospective West London First-Episode Psychosis study (WLFEP) [19,20]. Participants presented to secondary care services within the boroughs of Ealing, Hammersmith and Fulham, Wandsworth, Kingston, Richmond, Merton, Sutton and Hounslow, between 1998 and 2006. Ethical approval was obtained from local ethics committees of all boroughs included and written informed consent was obtained (RREC 3006). The study was therefore undertaken in accordance with the ethical standards defined in the 1964 Declaration of Helsinki and subsequent amendments therein.

Participants

Participants included for participation were aged 16 years or over, experiencing a first psychotic episode, had received fewer than 12 weeks antipsychotic medication and spoke sufficient English to facilitate assessment. Potential participants were screened using the World Health Organization Psychosis Screen [21] and diagnosed according to DSM-III [22] and DSM-IV criteria [23] by two psychiatric research nurses using the diagnostic module of the Diagnostic Interview for Psychosis [24]. See [17,25] for full details.

Data collection

Information was obtained, with informed consent, from participants' clinical records and clinical interview. Data gathered included basic demographics and symptom scores as well as performance on a number of clinical, cognitive and neuropsychological assessments. All researchers involved in data collection

For peer review only - http://bmjopen.bmj.com/site/about/guidelines.xhtml

received training to a high standard in the administration of these measures. All data presented were gathered at baseline / initial recruitment to the study.

Measures

Symptom dimension scores

Each participant's symptoms were characterised by three symptom dimension scores, which captured the severity of their positive, negative and disorganisation symptoms. These were derived from a second-order principal components analysis (PCA) of participants' individual item scores on the Scale for the Assessment of Positive Symptoms (SAPS) and the revised version of the Scale for the Assessment of Negative Symptoms (SANS-R) [26]; see [17,27] for further details.

Individual level covariates

Basic demographic information including age, gender and place of residence were gathered. Ethnicity data were not recorded. In addition, participants were assigned to one of five socioeconomic categories based on their occupation using the National Statistics Socio-Economic Classification system (NS-SEC) [28].

Neighbourhood level exposures

Each participant's postcode was used to identify their small area neighbourhood, based on the Census Area Statistics (CAS) ward in which they lived at first contact using databases produced by the ONS [29]. For each ward -henceforth referred to as a neighbourhood- a number of indices of urbanicity, deprivation, inequality and social capital were identified; unless specified otherwise, all these data were obtained from the 2001 census [29].

Following the work of others [30], population density (measured in people per hectare) was used as a proxy for urbanicity.

For peer review only - http://bmjopen.bmj.com/site/about/guidelines.xhtml

Two indices of deprivation were included in the study: (i) income deprivation (ID): the percentage of individuals who were living in a household with an income of less than 60% of the median, and (ii) the Index of Multiple Deprivation (IMD), an aggregate measure of deprivation comprised of 37 indicators, obtained from The English Indices of Deprivation (2004). Since these data were available for nested geographical areas smaller than the CAS ward –lower layer super output areas (LSOA)- neighbourhood level indices were calculated as the sum of composite LSOA values, weighted by their population size, as described previously [1].

In addition, it was also possible to derive indices of *inequality,* i.e. the dispersion of deprivation. For both the ID and IMD, a corresponding Gini coefficient was calculated for each neighbourhood (GINI-ID and GINI-IMD), based on the distribution of deprivation across its composite LSOAs, as described previously [1]. A Gini coefficient of zero represents perfect equality, whereas Gini coefficients approaching one indicate maximum inequality.

To calculate an index of social fragmentation (SFI), a composite of four separate 2001 census measures (Z-transformed and summed) were used, as described previously [31,32]: (i) the percentage of people aged 16 years or over and single; (ii) the percentage of households that were single-occupancy; (iii) the percentage of households that were rented; and (iv) the percentage of people who were mobile in the 12 months prior to the census date. As a proxy for social cohesion, voter turnout during the 2002 local elections (percentage of the electorate who cast valid ballots) was obtained for each neighbourhood [33] as described previously [6,13].

Ethnic segregation was estimated using the Index of Dissimilarity (IDS) [6]. IDS was calculated at the neighbourhood level, measuring the extent to which white and

For peer review only - http://bmjopen.bmj.com/site/about/guidelines.xhtml

black / minority / ethnic (BME) populations were segregated across Lower Output Areas (LSOAs) within each neighbourhood (IDS-BME). IDS scores ranged between 0 (no segregation) to 1 (total segregation). In addition, BME ethnic density was calculated (DEN-BME) as the proportion of BME individuals relative to the total population in each neighbourhood.

Statistical analyses

To determine the neighbourhood level predictors of psychotic symptoms, symptom dimension scores were regressed on predictors using multi-level linear regression. Prior to regression symptom dimension scores were transformed to minimize skew, using an optimal transformation (square-root, cube-root, logarithmic or inverse transformations) on the basis of which minimised skew to the greatest extent. The data were then Z-transformed to have a mean of zero and standard deviation of one, with extreme outliers (>3Z) discarded.

For all regression analyses a null multi-level model was run first to determine the proportion of variance explained by neighbourhood level random effects. Basic demographic information (age at assessment, sex and NS-SEC) were then added as potential *a priori* confounders with neighbourhood level predictors added subsequently using forward step-wise selection. These were only retained / added to the multivariable model if they significantly improved its fit ($p<0.05$; likelihood ratio test). NS-SEC and sex were coded as categorical variables, with ‘unemployed’ and ‘male’ set as reference levels.

To explore associations at the item level participants’ responses on the persecutory delusions (SAPS-D1) and (global) hallucinations (SAPS-H7) items of the SAPS were also regressed on predictors. Since the distribution of participants’ responses on these items could not be normalised through transformation they were re-coded

For peer review only - http://bmjopen.bmj.com/site/about/guidelines.xhtml

as binary variables, with scores of 0-2 coded as absent-to-mild and scores of 3-5 coded as moderate-to-severe, and analyses undertaken using multi-level logistic regression.

Regression analyses were undertaken using Stata (Version 14; StataCorp LP, College Station, TX).

Patient and public involvement statement

Patients and public were not involved in the design or analysis of this study.

Data availability

Data from this study are not openly available. The combination of demographic, socioeconomic and geographic information, measured at a fine-scale, coupled with the relative low incidence of psychosis would risk compromising participant anonymity.

Results

Sample

Unfortunately, information as to the number of potential participants that were evaluated, screened and eventually excluded was not routinely recorded throughout the study. Nonetheless, of the 379 participants for whom data were originally collected, 34 (8.97%) were discarded due to inappropriate residency: no fixed abode (n=5), incomplete postcode information (n=4) or resident beyond the study's catchment area (n=25). Of the remaining 345, complete symptom data were available for 335 participants (97.10%). Following transformation to minimise skew, eight outlying symptom component scores were removed. With respect to basic demographics, seven data-points were not coded at the time of data collection: age (n=2), gender (n=2), and occupation (n=3). Further, voter turn-out (SCI) was not

For peer review only - http://bmjopen.bmj.com/site/about/guidelines.xhtml

available for three neighbourhoods due to the existence of a single, unopposed representative. Taken together, these exclusions resulted in a complete core data-set of 319 participants, representing 84.17% of the original sample (see Table 1). Within this sample the median age was 24.16 years [inter-quartile range (IQR)=20-30.23], 65.83% were male, 57.05% were unemployed and 75.55% experienced non-affective psychosis. The median SAPS and SANS total global scores were 32 (IQR=23-45) and 18 (IQR=7-34), respectively. Table 1 also shows the duration of untreated psychosis (DUP, measured in weeks) in order to facilitate comparison with other studies (median=12; IQR=4-44). Whilst DUP was not included as an *a priori* confounder (since there is previous evidence this is not associated with neighbourhood level factors in London [34]), re-running our primary analyses with its inclusion did not impact upon the findings (data available from authors). Participants retained in the analyses did not differ from the original data-set on any of the individual or neighbourhood level variables (data available upon request).

For peer review only

For peer review only - http://bmjopen.bmj.com/site/about/guidelines.xhtml

Table 1 Demographic and clinical characteristics of the study sample. Statistics provided include the number and percentage of cases (N / %), the median and the inter-quartile range (IQR). Age=age at assessment; DUP=duration of untreated psychosis (in weeks); NS-SEC= National Statistics Socio-Economic Classification system; SAPS= Scales for the Assessment of Positive symptoms (global scores); SANS= Scales for the Assessment of Negative symptoms (global scores).

Variable	Level	N (%)	Median	IQR
Age	-	-	24.16	20-30.23
Gender	*Male*	210 (65.83)	-	-
	Female	109 (34.17)	-	-
NS-SEC	*Managerial and professional*	17 (5.33)	-	-
	Intermediate occupations	21 (6.58)	-	-
	Routine and manual	50 (15.67)	-	-
	Unemployed	182 (57.05)	-	-
	Students	49 (15.36)	-	-
Diagnosis	*Schizophrenia*	195 (61.13)	-	-
	Schizophreniform disorder	40 (12.54)	-	-
	Brief psychotic disorder	3 (0.94)	-	-
	Delusional disorder	3 (0.94)	-	-
	Schizoaffective disorder	42 (13.17)	-	-
	Bipolar disorder	20 (6.27)	-	-
	Major depression with psychotic features	10 (3.13)	-	-
	Not recorded	6 (1.88)	-	-
DUP	-	-	12	4-44
SAPS total	-	-	32	23-45
SANS total	-	-	18	7-34

For peer review only - http://bmjopen.bmj.com/site/about/guidelines.xhtml

Neighbourhood data

Data included were distributed across 113 neighbourhoods from 14 boroughs. The median number of LSOAs per neighbourhood was seven (IQR=6-8) and the median number of participants per neighbourhood was two (IQR=1-4). In order to characterise the neighbourhoods further the pattern of associations between key measures (Supplementary Table 1) were explored. Spearman's correlations (Supplementary Table 2) indicated that, at a corrected alpha of 0.002 (corrected for 21 comparisons), the more deprived neighbourhoods (high IMD) were significantly more densely populated ($p<0.001$), less unequal ($p<0.001$), more socially fragmented ($p<0.001$), less socially cohesive ($p<0.001$) and characterised by higher ethnic segregation ($p<0.01$) and a higher density of ethnic minorities ($p<0.001$). Further, more unequal neighbourhoods were significantly less densely populated ($p<0.001$), less deprived ($p<0.001$), less socially fragmented ($p<0.001$), more socially cohesive ($p<0.001$) and had a lower density of ethnic minorities ($p<0.001$).

Multi-level modelling of symptom components and symptom scores

Fixed effects

Analyses undertaken whilst controlling for age, gender and social class- ($\chi^2_{(7)}$=24.25, p=0.001) indicated that inequality (GINI-ID) was significantly associated with negative symptoms (Table 2 and Supplementary Table 3): higher inequality was associated with less severe symptoms (coefficient=-1.54, CI=-2.76, -0.33, p=0.01). This effect persisted after further control for other (positive and disorganisation) symptoms (coefficient=-1.66, CI=-2.86, -0.46, p<0.01) as well as absolute deprivation, i.e. ID or IMD (coefficient=-2.06, CI=-3.32,-0.8, p<0.01; coefficient=-2.06, CI=-3.32,-0.8, p<0.01). Further, ethnic segregation (IDS-BME) was associated with positive symptoms after controlling for basic demographics: higher segregation of ethnic minorities was associated with less severe positive

For peer review only - http://bmjopen.bmj.com/site/about/guidelines.xhtml

symptoms (coefficient=-2.36, CI=-4.2, -0.52, p=0.01; Table 2 and Supplementary Table 4). This effect also persisted after controlling for other symptoms (coefficient=-2.32, CI=-4.17, -0.48, p=0.01) as well as absolute deprivation, i.e. ID or IMD (coefficient=-2.2, CI=-4.09, -0.32, p=0.02; coefficient=-2.17, CI=-4.07,-0.28, p=0.03). However, no significant neighbourhood level predictors were found for disorganisation symptoms (Table 2 and Supplementary Table 5). Nor did any neighbourhood level predictor predict significant variance in paranoid delusions or global hallucinations (symptom level analyses; Supplementary Table 6).

Random effects

Neighbourhood level random effects did not explain significant levels of variance in symptom dimension or individual symptom scores, either before or after controlling for demographic variables: negative symptoms (<0.001%), positive symptoms (<0.001%), disorganisation symptoms (1.45%), paranoid delusions (5.35%) or global hallucinations (<0.001%) (all p-values>0.05).

For peer review only - http://bmjopen.bmj.com/site/about/guidelines.xhtml

Table 2 Multi-level modelling of symptom components (summary). Individual symptom dimensions derived from second-order principal component analysis were regressed on predictor variables using multi-level regression analyses. Data reported are from multivariate models that were run whilst controlling for basic demographic information (age, gender and NS-SEC) and other symptoms (e.g. negative and disorganisation symptoms for the positive symptoms). Neighbourhood level variables are in italics. Significant variables are in bold.

		Negative symptoms		Positive symptoms		Disorganisation symptoms	
		Fixed part of the model		Fixed part of the model		Fixed part of the model	
Predictor	Level	Coefficient (95% CIs)	Wald p value	Coefficient (95% CIs)	Wald p value	Coefficient (95% CIs)	Wald p value
Age		0.01 (-0.01, 0.02)	0.37	-0.01 (-0.02, 0)	0.12	-0.01 (-0.02, 0.01)	0.33
Gender	Female	-0.36 (-0.59, -0.12)	**<0.01**	0.06 (-0.18, 0.3)	0.63	-0.23 (-0.47, 0.01)	0.06
NS-SEC	Managerial	-0.49 (-0.98, 0)	0.05	0.03 (-0.47, 0.53)	0.91	-0.22 (-0.73, 0.29)	0.39
	Intermediate	-0.23 (-0.67, 0.22)	0.32	0.49 (0.05, 0.94)	**0.03**	-0.08 (-0.54, 0.37)	0.72
	Routine	-0.35 (-0.65, -0.04)	**0.03**	0.16 (-0.15, 0.47)	0.32	-0.26 (-0.57, 0.05)	0.1
	Student	0.09 (-0.23, 0.4)	0.59	0.07 (-0.25, 0.39)	0.67	-0.21 (-0.53, 0.12)	0.21
Pop Den		-	-	-	-	-	-
ID		-	-	-	-		
IMD		-	-	-	-	-	-
GINI-ID		-1.66 (-2.86, -0.46)	**<0.01**	-	-	-	-
GINI-IMD		**-**	**-**	-	-	-	-
SFI		-	-	-	-	-	-
SCI		-	-	-	-	-	-
IDS-BME		-	-	-2.32 (-4.17, -0.48)	**0.01**	-	-
DEN-BME		-	-	-	-	-	-

For peer review only

Discussion

Fixed effects analyses revealed that each symptom dimension (positive, negative and disorganisation) was associated with a distinct pattern of neighbourhood level risk factors; thus, higher levels of inequality were associated with lower negative symptoms, and greater ethnic segregation was associated with lower positive symptoms. However, contrary to our primary hypotheses, there was no evidence to suggest that the positive symptoms of psychosis were elevated in areas scoring highly on classical indicators of environmental adversity or socioeconomic deprivation (e.g. population density, income deprivation or inequality), either at the level of the symptom cluster, or with respect to persecutory delusions or hallucinations specifically.

Nonetheless, the finding that participants living in neighbourhoods characterised by highly segregated BME communities exhibited *less* severe positive symptoms is interesting, since the index of dissimilarity speaks to the richness of the social environment. Thus, whilst a high index of dissimilarity indicates high segregation *between* populations, it implies reduced fragmentation *within* a given ethnic group, i.e. a high potential for bonding social capital [35,36], with potential protective effects against the positive symptoms of psychosis [37]. This is consistent with a previous study undertaken in South East London, which reported a lower incidence of psychosis in areas characterised by higher (BME) ethnic segregation [6], as well as research showing that the risk of psychosis diminishes as the proportion of one's own ethnic group increases within a neighbourhood [37–40].

The finding that participants living in neighbourhoods characterised by higher inequality (GINI-ID) exhibited less severe negative symptoms is inconsistent with the Income Inequality Hypothesis (IIH) [41], which posits that highly unequal

For peer review only - http://bmjopen.bmj.com/site/about/guidelines.xhtml

neighbourhoods are characterised by poorer health outcomes. However, the empirical findings on the association between income inequality and mental health are not clear cut. A recent qualitative synthesis identified 27 relevant studies that explored this issue [42], nine of which met inclusion criteria for a meta-analysis; of these, only one found a positive association between higher inequality and poorer mental health, whilst six reported mixed results, and two found no significant effects. The authors concluded that the extant literature in the field is characterised by small effect sizes and a high level of heterogeneity in findings. See [43,44] also.

The association between inequality and psychosis -rather than mental health in general- is also not clear-cut. For example, whilst no association was seen between income inequality and psychotic *symptoms* in a study of FEP undertaken in South East London and Nottinghamshire [13], links between inequality and psychosis *incidence* or *prevalence* have been shown using sample sizes comparable to our own [1,4,45]. It is not inconceivable, however, that inequality and/or deprivation might be characterised by relatively independent (or even inverted) patterns of association with psychosis incidence and psychotic symptom severity. For example, one might imagine how in a wealthy neighbourhood, in which health and social services are well resourced (for detection as well as treatment), the recorded incidence of psychosis might be elevated but the severity of symptoms in those individuals reduced.

Another potential explanation for the unexpected finding of an association between higher inequality and lower negative symptoms scores resides in the socio-political geography of the West London region examined. According to the Social Capital Hypothesis [46] areas that are relatively more unequal, i.e. defined by a higher Gini coefficient, tend to have worse health outcomes because they are characterised by lower levels of interpersonal trust and a lack of a sense of shared community (i.e.

For peer review only - http://bmjopen.bmj.com/site/about/guidelines.xhtml

low social capital and high social fragmentation). Thus, poor social cohesion / social capital is thought to mediate the negative effects of inequality on health [47]. However, within the WLFEP data-set the inverse was true (Table 2): more unequal neighbourhoods were in fact *more* socially cohesive, characterised by extremes of affluence (rather than deprivation) and a relative abundance of social capital. Although a direct association was not seen between social cohesion and psychotic symptoms in our study, it is possible that more sensitive indices of social capital, or measures that tap into different facets of this complex construct [48], might uncover such an association [49] .

Finally, the reported association between higher inequality and lower negative symptoms might be explained by the Mixed Neighbourhood Hypothesis (MNH) [50–52]. According to the MNH, the mixing of individuals from different socioeconomic backgrounds within areas of high inequality may be protective against cultures of crime, substance use, joblessness and a lack of social opportunity that can become endemic to areas of homogenous deprivation. Further, the wealthy may also bring higher investment in local infrastructure and resources. Arguably, however, despite considerable political interest in the area, particularly in the US, reviews of the literature have typically failed to find convincing evidence for the MNH [17,42] or the benefits of mixed housing schemes that are linked to the theory [53].

With respect to the limitations of the study, there are several. First, its cross-sectional design precludes inferences about causality. Second, the use of national census data from a single time-point (2001) in conjunction with participant data collected across a broad temporal window (1998-2006) is likely to have increased measurement error, and therefore, potentially reduced the chance of finding a significant effect (inflation of type II error). Nonetheless, this could not be avoided since national census data are only collected every ten years.

For peer review only

With respect to the variables available for analysis, whilst ethnicity was controlled for at the ward- level, individual- level ethnicity was not collected. Consequently, individual- level ethnicity may have confounded some of the effects of ward- level indices, particularly those relating directly to ethnicity, e.g. the effect of ethnic segregation on positive symptoms. Thus, there is ample evidence that the incidence of psychotic disorders is elevated in migrant and minority ethnic populations, an effect that seems to persist even after controlling for individual- level SES [54]. Further, psychotic symptoms may also be elevated in individuals from these populations [55]. However, there is evidence to suggest that ethnicity does not predict differences in psychosis symptom dimension scores; see [13] for example.

The use of voter turnout as a proxy for social cohesion has some limitations. Whilst there is evidence to suggest that voter turnout does correlate with self-reported interpersonal and societal trust, e.g. [56], social cohesion is a complex and multi-faceted construct, and as such, is unlikely to be fully captured by a sole crude measure, i.e. it may have limited content validity; see [36]. Second, voter turnout, by definition, cannot represent individuals who are denied access to the electoral role, for example, individuals under the voting age (<16years), non-citizen migrants, refugees and asylum seekers. This is particularly relevant to the field of psychosis research, since migrant groups have an elevated risk of experiencing psychotic symptoms; see [57] for a review. Despite these limitations however, voter turnout has been highlighted as a headline measure of civic and political participation by the Organisation for Economic Co-operation and Development (OECD), has been included in the Office for National Statistics' National Well-being Wheel of Measures, and further, has been put forward as a sustainable development indicator by the Eurostat / OECD / UNECE Task Force on Measuring Sustainable Development; see [58] for further details.

For peer review only - http://bmjopen.bmj.com/site/about/guidelines.xhtml

Finally, since our sample was characterised by relatively few participants per neighbourhood we were likely under-powered to detect random effects, particularly in view of the fact that estimates of random effects are comparatively unreliable and biased towards under-estimation [59]. Nonetheless, consistent with our *a priori* aims, neighbourhood covariates (i.e. fixed effects) were still modelled and significant associations found.

In conclusion, the findings reported contribute to a growing body of evidence that highlights the importance of social and socioeconomic factors in the expression of psychosis symptoms. Further, they suggest that at least some of the association between psychosis and the environment may be operating at the level of symptoms, with specific environmental predictors linked to specific symptoms or symptoms clusters. Arguably, this lends support to current calls in both clinical [60] and research [61] practice for dimension based models of psychosis [62–64], a debate that is also echoed more broadly in the field of general psychopathology [65,66]. Finally, the findings also highlight the need for longitudinal research that can begin to elucidate the underlying mechanisms that link the environment to symptom expression, in addition to establishing the direction of causality.

For peer review only

Author contributions: EJ and TB designed and oversaw the West London First Episode Psychosis Study (WLFEP). SM, IH and VH tested participants. VH and MT designed the current study. MT undertook all analyses and wrote the manuscript. JK provided advice and support on statistical analyses. All authors commented on -and contributed to- the final version of the manuscript.

For peer review only - http://bmjopen.bmj.com/site/about/guidelines.xhtml

References

1 Kirkbride JB, Jones PB, Ullrich S, *et al.* Social deprivation, inequality, and the neighborhood-level incidence of psychotic syndromes in East London. *Schizophr Bull* 2014;**40**:169–80.http://ovidsp.ovid.com/ovidweb.cgi?T=JS&PAGE=reference&D=psyc11&NEWS=N&AN=2014-05521-022

2 Heinz A, Deserno L, Reininghaus U. Urbanicity, social adversity and psychosis. *World Psychiatry* 2013;**12**:187–97. doi:10.1002/wps.20056

3 O'Donoghue B, Roche E, Lane A. Neighbourhood level social deprivation and the risk of psychotic disorders: a systematic review. *Soc Psychiatry Psychiatr Epidemiol* 2016;**51**:941–50. doi:10.1007/s00127-016-1233-4

4 Burns JK, Esterhuizen T. Poverty, inequality and the treated incidence of first-episode psychosis: an ecological study from South Africa. *Soc Psychiatry Psychiatr Epidemiol* 2008;**43**:331–5. doi:10.1007/s00127-008-0308-2

5 Burns JK, Tomita A, Kapadia AS. Income inequality and schizophrenia: increased schizophrenia incidence in countries with high levels of income inequality. *Int J Soc Psychiatry* 2014;**60**:185–96. doi:10.1177/0020764013481426

6 Kirkbride JB, Morgan C, Fearon P, *et al.* Neighbourhood-level effects on psychoses: re-examining the role of context. *Psychol Med* 2007;**37**:1413–25. doi:10.1017/S0033291707000499

7 Bentall RP, de Sousa P, Varese F, *et al.* From adversity to psychosis: pathways and mechanisms from specific adversities to specific symptoms. *Soc Psychiatry Psychiatr Epidemiol* 2014;**49**:1011–22. doi:10.1007/s00127-014-0914-0

8 Ellett L, Freeman D, Garety P. The psychological effect of an urban environment on individuals with persecutory delusions: the Camberwell walk

For peer review only - http://bmjopen.bmj.com/site/about/guidelines.xhtml

study. *Schizophr Res* 2008;**99**:77–84. doi:10.1016/j.schres.2007.10.027

9 Veling W, Pot-Kolder R, Counotte J, *et al.* Environmental Social Stress, Paranoia and Psychosis Liability: A Virtual Reality Study. *Schizophr Bull* 2016;**42**:1363–71. doi:10.1093/schbul/sbw031

10 Bentall RP, Corcoran R, Howard R, *et al.* Persecutory delusions: a review and theoretical integration. *Clin Psychol Rev* 2001;**21**:1143–92.http://www.ncbi.nlm.nih.gov/pubmed/11702511 (accessed 14 Nov 2018).

11 Freeman D, Garety PA, Kuipers E, *et al.* A cognitive model of persecutory delusions. *Br J Clin Psychol* 2002;**41**:331–47.http://www.ncbi.nlm.nih.gov/pubmed/12437789 (accessed 14 Nov 2018).

12 Mirowsky J, Ross CE. Paranoia and the structure of powerlessness. *Am Sociol Rev* 1983;**48**:228–39.http://www.ncbi.nlm.nih.gov/pubmed/6859680 (accessed 14 Nov 2018).

13 Oher FJ, Demjaha A, Jackson D, *et al.* The effect of the environment on symptom dimensions in the first episode of psychosis: a multilevel study. *Psychol Med* 2014;**44**:2419–30. doi:10.1017/S0033291713003188

14 Wickham S, Taylor P, Shevlin M, *et al.* The Impact of Social Deprivation on Paranoia, Hallucinations, Mania and Depression: The Role of Discrimination Social Support, Stress and Trust. *PLoS One* 2014;**9**. doi:10.1371/journal.pone.0105140

15 Johnson SL, Wibbels E, Wilkinson R. Economic inequality is related to cross-national prevalence of psychotic symptoms. Soc. Psychiatry Psychiatr. Epidemiol. 2015;:No-Specified.http://ovidsp.ovid.com/ovidweb.cgi?T=JS&PAGE=reference&D=psyc11&NEWS=N&AN=2015-42803-001

16 Newbury J, Arseneault L, Caspi A, *et al.* Why Are Children in Urban Neighborhoods at Increased Risk for Psychotic Symptoms? Findings From a UK Longitudinal Cohort Study. *Schizophr Bull* 2016;**42**:1372–83.

For peer review only - http://bmjopen.bmj.com/site/about/guidelines.xhtml

doi:10.1093/schbul/sbw052

17 Tibber MS, Kirkbride JB, Joyce EM, *et al.* The component structure of the scales for the assessment of positive and negative symptoms in first-episode psychosis and its dependence on variations in analytic methods. *Psychiatry Res* 2018;**270**:869–79. doi:10.1016/J.PSYCHRES.2018.10.046

18 Johnson SL, Wibbels E, Wilkinson R. Economic inequality is related to cross-national prevalence of psychotic symptoms. *Soc Psychiatry Psychiatr Epidemiol* 2015;**50**:1799–807.http://ovidsp.ovid.com/ovidweb.cgi?T=JS&PAGE=reference&D=medl&NEWS=N&AN=26370212

19 Barnes TR, Hutton SB, Chapman MJ, *et al.* West London first-episode study of schizophrenia. Clinical correlates of duration of untreated psychosis. *Br J Psychiatry* 2000;**177**:207–11.http://www.ncbi.nlm.nih.gov/pubmed/11040879 (accessed 6 Mar 2017).

20 Joyce EM, Hutton SB, Mutsatsa SH, *et al.* Cognitive heterogeneity in first-episode schizophrenia. *Br J Psychiatry* 2005;**187**:516–22. doi:10.1192/bjp.187.6.516

21 Jablensky A, Sartorius N, Ernberg G, *et al.* Schizophrenia: manifestations, incidence and course in different cultures. A World Health Organization ten-country study. *Psychol Med Monogr Suppl* 1992;**20**:1–97.http://www.ncbi.nlm.nih.gov/pubmed/1565705 (accessed 29 May 2017).

22 American Psychiatric Association. *Diagnostic and Statistical Manual of Mental Disorders - Third Edition*. III. Washington, DC: : American Psychiatric Association 1980.

23 American Psychiatric Association. *Diagnostic and Statistical Manual of Mental Disorders - Fourth Edition*. 4th ed. Washington, DC: : American Psychiatric Association 2000.

24 Castle DJ, Jablensky A, McGrath JJ, *et al.* The diagnostic interview for

For peer review only - http://bmjopen.bmj.com/site/about/guidelines.xhtml

psychoses (DIP): development, reliability and applications. *Psychol Med* 2006;**36**:69–80. doi:10.1017/S0033291705005969

25 Huddy VC, Hodgson TL, Kapasi M, *et al.* Gaze strategies during planning in first-episode psychosis. *J Abnorm Psychol* 2007;**116**:589–98. doi:10.1037/0021-843X.116.3.589

26 Andreasen N. Methods for assessing positive and negative symptoms. In: Andreasen N, ed. *Schizophrenia : Positive and Negative Symptoms and Syndromes. Modern Problems in Pharmacopsychiatry*. Basel: : Karger 1990. 73–85.

27 Peralta V, Cuesta MJ. Dimensional structure of psychotic symptoms: an item-level analysis of SAPS and SANS symptoms in psychotic disorders. *Schizophr Res* 1999;**38**:13–26.http://www.ncbi.nlm.nih.gov/pubmed/10427607 (accessed 26 Jul 2015).

28 Rose D, Pevalin D. The National Statistics Socio-economic Classification: Origins, Development and Use. Norwich: 2005.

29 Office for National Statistics. Nomis - official labour market statistics - 2001 census data. https://www.nomisweb.co.uk

30 Kaymaz N, Krabbendam L, de Graaf R, *et al.* Evidence that the urban environment specifically impacts on the psychotic but not the affective dimension of bipolar disorder. *Soc Psychiatry Psychiatr Epidemiol* 2006;**41**:679–85. doi:10.1007/s00127-006-0086-7

31 Congdon P. Suicide and Parasuicide in London: A Small-area Study. *Urban Stud* 1996;**33**:137–58. doi:10.1080/00420989650012194

32 Allardyce J, Gilmour H, Atkinson JM, *et al.* Social fragmentation, deprivation and urbanicity: relation to first-admission rates for psychoses. *Br J Psychiatry* Published Online First: 1 November 2005. doi:10.1192/bjp.187.5.401 <http://dx.doi.org/10.1192/bjp.187.5.401>)

33 Rallings C, Thrasher M. Local Elections Handbook 2002. Plymouth: 2002.

For peer review only - http://bmjopen.bmj.com/site/about/guidelines.xhtml

http://www.electionscentre.co.uk/?page_id=3684

34 Kirkbride JB, Lunn DJ, Morgan C, *et al.* Examining evidence for neighbourhood variation in the duration of untreated psychosis. *Health Place* 2010;**16**:219–25. doi:10.1016/j.healthplace.2009.09.013

35 Becares L, Nazroo J. Social capital, ethnic density and mental health among ethnic minority people in England: a mixed-methods study. *Ethn Health* 2013;**18**:544–62. doi:10.1080/13557858.2013.828831

36 Orford J. Two Concepts for Understanding Inequalities by Position and Place - social capital and sense of community. In: *Community Psychology - Challenges, Controversies and Emerging Consensus*. Chichester: : John Wiley & Sons Ltd. 2008. 163–81.

37 Das-Munshi J, Bécares L, Boydell JE, *et al.* Ethnic density as a buffer for psychotic experiences: findings from a national survey (EMPIRIC). *Br J Psychiatry* 2012;**201**.http://bjp.rcpsych.org/content/201/4/282 (accessed 19 May 2017).

38 Boydell J, van Os J, McKenzie K, *et al.* Incidence of schizophrenia in ethnic minorities in London: ecological study into interactions with environment. *BMJ* 2001;**323**:1336–8.http://www.ncbi.nlm.nih.gov/pubmed/11739218 (accessed 20 Mar 2017).

39 Schofield P, Thygesen M, Das-Munshi J, *et al.* Ethnic density, urbanicity and psychosis risk for migrant groups – A population cohort study. *Schizophr Res* 2017;**190**:82–7. doi:10.1016/j.schres.2017.03.032

40 Veling W, Susser E, van Os J, *et al.* Ethnic Density of Neighborhoods and Incidence of Psychotic Disorders Among Immigrants. *Am J Psychiatry* 2008;**165**:66–73. doi:10.1176/appi.ajp.2007.07030423

41 Wilkinson RG, Pickett K. *The Spirit Level*. London: : The Penguin Group 2009.

42 Ribeiro WS, Bauer A, Andrade MCR, *et al.* Income inequality and mental

For peer review only - http://bmjopen.bmj.com/site/about/guidelines.xhtml

illness-related morbidity and resilience: a systematic review and meta-analysis. *The Lancet Psychiatry* 2017;**4**:554–62. doi:10.1016/S2215-0366(17)30159-1

43 Wilkinson R, Pickett K. *The Inner Level - How More Equal Societies Reduce Stress, Restore Sanity and Improve Everyone's Wellbeing*. First. St Ives: : Penguin Random House 2018.

44 Tibber M. *Individual- and neighbourhood-level predictors of psychotic symptom dimensions in West London.* 2017.http://discovery.ucl.ac.uk/1575465/

45 Boydell J, van Os J, McKenzie K, *et al.* The association of inequality with the incidence of schizophrenia - An ecological study. *Soc Psychiatry Psychiatr Epidemiol* 2004;**39**:597–9. doi:10.1007/s00127-004-0789-6

46 McKenzie K, Whitely R, Weich S. Social capital and mental health. *Br J Psychiatry* 2002;**181**.

47 Kragten N, Rözer J. The Income Inequality Hypothesis Revisited: Assessing the Hypothesis Using Four Methodological Approaches. *Soc Indic Res* 2017;**131**:1015–33. doi:10.1007/s11205-016-1283-8

48 Siegler V. Measuring Social Capital. 2014.

49 Clough-Gorr KM, Egger M, Spoerri A. A Swiss paradox? Higher income inequality of municipalities is associated with lower mortality in Switzerland. *Eur J Epidemiol* 2015;**30**:627–36. doi:10.1007/s10654-015-9987-7

50 Manley D, VanHam M, Doherty J. Social mixing as a cure for negative neighbourhood effects: evidence based policy or urban myth? In: Bridge G, Butler T, Lees L, eds. *Mixed Communities: gentrification by stealth?* Bristol: : The Policy Press 2012. 1–17.

51 Musterd S, Andersson R. Housing Mix, Social Mix, and Social Opportunities. *Urban Aff Rev* 2005;**40**:761–90. doi:10.1177/1078087405276006

52 Ostendorf W, Musterd S, De Vos S. Social Mix and the Neighbourhood

For peer review only

For peer review only - http://bmjopen.bmj.com/site/about/guidelines.xhtml

Effect. Policy Ambitions and Empirical Evidence. *Hous Stud* 2001;**16**:371–80. doi:10.1080/02673030120049724

53 Melis G, Marra G, Gelormino E. Housing and Social Mix - Equity Action. 2013. http://www.health-inequalities.eu/wp-content/uploads/2016/05/Literature-Review-housing_social-mix-final.pdf

54 Kirkbride J, Errazuriz A, Croudace T, *et al.* Systematic Review of the Incidence and Prevalence of Schizophrenia and Other Psychoses in England. London, England: 2012. http://www.psychiatry.cam.ac.uk/files/2014/05/Final-report-v1.05-Jan-12.pdf

55 King M, Nazroo J, Weich S, *et al.* Psychotic symptoms in the general population of England. *Soc Psychiatry Psychiatr Epidemiol* 2005;**40**:375–81. doi:10.1007/s00127-005-0900-7

56 Arora M, Mendoza MA, Kim H. A Cross-National Study of the Effects of Social Capital on Voter Turnout. In: *Midwest Political Science Association Annual Conference*. 2016. 1–23.

57 Parrett NS, Mason OJ. Refugees and psychosis: A review of the literature. *Psychosis* 2010;**2**:111–21. doi:10.1080/17522430903219196

58 Siegler V. Measuring Social Capital in the UK. 2014.

59 Bryan ML, Jenkins SP. Multilevel Modelling of Country Effects: A Cautionary Tale. *Eur Sociol Rev* 2016;**32**:3–22. doi:10.1093/esr/jcv059

60 Parker GF. DSM-5 and Psychotic and Mood Disorders. *J Am Acad Psychiatry Law Online* 2014;**42**.http://jaapl.org/content/42/2/182 (accessed 19 May 2017).

61 Morris SE, Cuthbert BN. Research Domain Criteria: cognitive systems, neural circuits, and dimensions of behavior. *Dialogues Clin Neurosci* 2012;**14**:29–37.http://www.ncbi.nlm.nih.gov/pubmed/22577302 (accessed 16 May 2017).

62 Allardyce J, McCreadie RG, Morrison G, *et al.* Do symptom dimensions or categorical diagnoses best discriminate between known risk factors for

For peer review only - http://bmjopen.bmj.com/site/about/guidelines.xhtml

psychosis? *Soc Psychiatry Psychiatr Epidemiol* 2007;**42**:429–37. doi:10.1007/s00127-007-0179-y

63 Van Os J, Gilvarry C, Bale R, *et al.* A comparison of the utility of dimensional and categorical representations of psychosis. UK700 Group. *Psychol Med* 1999;**29**:595–606.http://www.ncbi.nlm.nih.gov/pubmed/10405080 (accessed 11 Aug 2015).

64 Van Os J. The transdiagnostic dimension of psychosis: implications for psychiatric nosology and research. *Shanghai Arch psychiatry* 2015;**27**:82–6. doi:10.11919/j.issn.1002-0829.215041

65 Lahey BB, Zald DH, Perkins SF, *et al.* Measuring the hierarchical general factor model of psychopathology in young adults. *Int J Methods Psychiatr Res* 2018;**27**:e1593. doi:10.1002/mpr.1593

66 Kotov R, Krueger RF, Watson D. A paradigm shift in psychiatric classification: the Hierarchical Taxonomy Of Psychopathology (HiTOP). *World Psychiatry* 2018;**17**:24–5. doi:10.1002/wps.20478

For peer review only

Supplementary Table 1 Key statistics for neighbourhood level variables (N=113 unique neighbourhoods included in the analysis). Statistics provided include the number of entries (N), the median and the interquartile range (IQR). The same data are also shown for all neighbourhoods in England (N=7932) for comparison, or else, all neighbourhoods with more than one composite LSOA (N=6685) where this was required to calculate an index (e.g. GINI-ID). Population density=general population density in people per hectare; ID=income deprivation; IMD=index of multiple deprivation; GINI-ID=Gini coefficient based on income deprivation; GINI-IMD=Gini coefficient based on index of multiple deprivation; SFI=social fragmentation index; SCI=social cohesion index; IDS-BME=index of dissimilarity for BME versus white populations; DEN-BME=BME ethnic density (people per hectare).

	Study data		National data	
	N	Median (IQR)	N	Median (IQR)
Pop density	113	63.91 (40.45-86.68)	7932	12.01 (0.02-236.05)
ID	113	11 (7.09-18)	7932	8.66 (1.03-61.71)
IMD	113	16.65 (10.2-24.33)	7932	14.68 (1.1-79.81)
GINI-ID	113	0.24 (0.18-0.3)	6685	0.21 (0-0.57)
GINI-IMD	113	0.17 (0.12-0.21)	6685	0.17 (0-0.65)
SFI	113	5.1 (1.76-7.69)	7932	-0.83 (-6.7-22.48)
SCI	113	33 (28.45-38.05)	7932	33.3
IDS-BME	113	0.13 (0.11-0.19)	6685	0.21 (0-0.79)
DEN-BME	113	0.17 (0.1-0.28)	7932	0.01 (0-0.86)

Supplementary Table 2 Spearman's correlations between neighbourhood level variables (n=113 unique neighbourhoods). Provided statistics include Spearman's Rho and p value (in brackets). Significant correlations at an alpha of 0.002 (corrected for 21 comparisons) are shown in bold. POP DENS=population density; IMD=index of multiple deprivation; GINI-IMD=Gini coefficient calculated for the index of multiple deprivation; SFI=social fragmentation index; SCI=social cohesion index (voter turnout); IDS-BME=index of dissimilarity for BME versus white populations; DEN-BME=BME ethnic density.

	IMD	GINI -IMD	SFI	SCI	IDS-BME	DEN-BME
POP DENS	**0.38 (<0.001)**	**-0.31 (<0.001)**	**0.65 (<0.001)**	**-0.42 (<0.001)**	0.24 (<0.01)	**0.31 (<0.001)**
IMD		**-0.38 (<0.001)**	**0.32 (<0.001)**	**-0.62 (<0.001)**	**0.29 (<0.01)**	**0.75 (<0.001)**
GINI-IMD		-	**-0.32 (<0.001)**	**0.45 (<0.001)**	0.05 (0.59)	**-0.31 (<0.001)**
SFI			-	**-0.41 (<0.001)**	0.09 (0.33)	0.05 (0.6)
SCI				-	-0.18 (0.06)	**-0.59 (<0.001)**
IDS-BME					-	**0.3 (<0.01)**

Supplementary Table 3 Negative symptoms, derived from second- order principle components analysis of individual item scores, regressed on predictor variables. The top line of the table indicates a random effects only model. Columns three and four show the results of a series of univariate analyses, examining the effects of adding individual predictors to a model that controls for core demographics only (age, gender and socioeconomic status). Model one shows the results of a multivariate analysis that includes all variables selected for inclusion. Model two is identical to model one except that other symptoms (positive and disorganisation symptoms) are also controlled for. Neighbourhood level variables are in italics. Significant p values are in bold.

		Univariate (controlling for basic demographics only)					Model 1 – Multivariate (basic)		Model 2 – Multivariate (controlling for other Sxs also)	
		Fixed part of model		Random part of model			Fixed part of the model		Fixed part of the model	
Predictor	Level	Coefficient (95% CIs)	Wald p value	Neighbourhood level variance	Chi-squared (1 df)	Chi-squared p value	Coefficient (95% CIs)	Wald p value	Coefficient (95% CIs)	Wald p value
Random only		-	-	0	0	1	-	-	-	-
Age		0.01 (-0.01, 0.02)	0.45	0	0	1	0.01 (-0.01, 0.02)	0.33	0.01 (-0.01, 0.02)	0.37
Gender	Female	-0.32 (-0.56, -0.08)	**<0.01**	0	0	1	-0.33 (-0.56, -0.09)	**<0.01**	-0.36 (-0.59, -0.12)	**<0.01**
NS-SEC	Managerial	-0.51 (-1.01, -0.01)	0.05	0	0	1	-0.47 (-0.97, 0.03)	0.06	-0.49 (-0.98, 0)	0.05
	Intermediate	-0.26 (-0.71, 0.19)	0.26	0	0	1	-0.2 (-0.65, 0.24)	0.37	-0.23 (-0.67, 0.22)	0.32
	Routine	-0.33 (-0.64, -0.02)	**0.03**	0	0	1	-0.31 (-0.61, -0.01)	**<0.05**	-0.35 (-0.65, -0.04)	**0.03**
	Student	0.08 (-0.24, 0.41)	0.61	0	0	1	0.12 (-0.2, 0.44)	0.46	0.09 (-0.23, 0.4)	0.59
Pop Den		0 (0, 0)	0.35	0	0	1	-	-	-	-
ID		-0.01 (-0.02, 0.01)	0.25	0	0	1	-	-	-	-
IMD		-0.01 (-0.02, 0.01)	0.39	0	0	1	-	-	-	-
GINI-ID		-1.54 (-2.76, -0.33)	**0.01**	0	0	1	-1.54 (-2.76, -0.33)	**0.01**	-1.66 (-2.86, -0.46)	**<0.01**
GINI-IMD		-1.42 (-2.93, 0.09)	0.07	0	0	1	-	-	-	-
SFI		-0.02 (-0.05, 0.01)	0.24	0	0	1	-	-	-	-
SCI		0 (-0.02, 0.02)	0.77	0	0	1	-	-	-	-
IDS-BME		-1.51 (-3.35, 0.33)	0.11	0	0	1	-	-	-	-
DEN-BME		0.33 (-0.27, 0.93)	0.28	0	0	1	-	-	-	-

Supplementary Table 4 Positive symptoms, derived from second- order principle components analysis of individual item scores, regressed on predictor variables. The top line of the table indicates a random effects only model. Columns three and four show the results of a series of univariate analyses, examining the effects of adding individual predictors to a model that controls for core demographics only (age, gender and socioeconomic status). Model one shows the results of a multivariate analysis that includes all variables selected for inclusion. Model two is identical to model one except that other symptoms (negative and disorganisation symptoms) are also controlled for. Neighbourhood level variables are in italics. Significant p values are in bold.

		Univariate (controlling for basic demographics only)					Model 1 –Multivariate (basic)		Model 2 –Multivariate (controlling for other Sxs also)	
		Fixed part of model		Random part of model			Fixed part of the model		Fixed part of the model	
Predictor	Level	Coefficient (95% CIs)	Wald p value	Neighbourhood level variance	Chi-squared (1 df)	Chi-squared p value	Coefficient (95% CIs)	Wald p value	Coefficient (95% CIs)	Wald p value
Random only		-	-	0	0	1	-	-	-	-
Age		-0.01 (-0.02, 0)	0.11	0	0	1	-0.01 (-0.02, 0)	0.12	-0.01 (-0.02, 0)	0.12
Gender	Female	0.04 (-0.2, 0.28)	0.75	0	0	1	0.05 (-0.19, 0.28)	0.7	0.06 (-0.18, 0.3)	0.63
NS-SEC	Managerial	-0.04 (-0.55, 0.46)	0.86	0	0	1	0.01 (-0.49, 0.51)	0.96	0.03 (-0.47, 0.53)	0.91
	Intermediate	0.47 (0.01, 0.92)	**0.04**	0	0	1	0.49 (0.04, 0.93)	**0.03**	0.49 (0.05, 0.94)	**0.03**
	Routine	0.15 (-0.16, 0.46)	0.35	0	0	1	0.14 (-0.16, 0.45)	0.35	0.16 (-0.15, 0.47)	0.32
	Student	0.11 (-0.22, 0.43)	0.51	0	0	1	0.07 (-0.25, 0.39)	0.68	0.07 (-0.25, 0.39)	0.67
Pop Den		0 (0, 0)	0.49	0	0	1	-	-	-	-
ID		-0.01 (-0.02, 0.01)	0.27	0	0	1	-	-	-	-
IMD		-0.01 (-0.02, 0)	0.22	0	0	1	-	-	-	-
GINI-ID		0.42 (-0.81, 1.65)	0.5	0	0	1	-	-	-	-
GINI-IMD		0.58 (-0.94, 2.1)	0.46	0	0	1	-	-	-	-
SFI		0.01 (-0.02, 0.04)	0.72	0	0	1	-	-	-	-
SCI		0.02 (0, 0.04)	0.08	0	0	1	-	-	-	-
IDS-BME		-2.36 (-4.2, -0.52)	**0.01**	0	0	1	-2.36 (-4.2, -0.52)	**0.01**	-2.32 (-4.17, -0.48)	**0.01**
DEN-BME		-0.55 (-1.15, 0.05)	0.07	0	0	1	-	-	-	-

For peer review only

Supplementary Table 5 Disorganisation symptoms, derived from second- order principle components analysis of individual item scores, regressed on predictor variables. The top line of the table indicates a random effects only model. Columns three and four show the results of a series of univariate analyses, examining the effects of adding individual predictors to a model that controls for core demographics only (age, gender and socioeconomic status). Model one shows the results of a multivariate analysis that includes all variables selected for inclusion. Model two is identical to model one except that other symptoms (negative and positive symptoms) are also controlled for. Neighbourhood level variables are in italics. Significant p values are in bold.

		Univariate (controlling for basic demographics only)					Model 1 –Multivariate (basic)		Model 2 –Multivariate (controlling for other Sxs also)	
		Fixed part of model		Random part of model			Fixed part of the model		Fixed part of the model	
Predictor	Level	Coefficient (95% CIs)	Wald p value	Neighbourhood level variance	Chi-squared (1 df)	Chi-squared p value	Coefficient (95% CIs)	Wald p value	Coefficient (95% CIs)	Wald p value
Random only		-	-	0.45	0.01	0.46	-	-	-	-
Age		-0.01 (-0.02, 0.01)	0.27	1.45	0.01	0.38	-0.01 (-0.02, 0.01)	0.27	-0.01 (-0.02, 0.01)	0.33
Gender	*Female*	-0.18 (-0.42, 0.06)	0.14	1.45	0.01	0.38	-0.18 (-0.42, 0.06)	0.14	-0.23 (-0.47, 0.01)	0.06
NS-SEC	*Managerial*	-0.15 (-0.65, 0.36)	0.57	1.45	0.01	0.38	-0.15 (-0.65, 0.36)	0.57	-0.22 (-0.73, 0.29)	0.39
	Intermediate	-0.04 (-0.5, 0.42)	0.86	1.45	0.01	0.38	-0.04 (-0.5, 0.42)	0.86	-0.08 (-0.54, 0.37)	0.72
	Routine	-0.21 (-0.52, 0.1)	0.19	1.45	0.01	0.38	-0.21 (-0.52, 0.1)	0.19	-0.26 (-0.57, 0.05)	0.1
	Student	-0.22 (-0.54, 0.11)	0.19	1.45	0.01	0.38	-0.22 (-0.54, 0.11)	0.19	-0.21 (-0.53, 0.12)	0.21
Pop Den		0 (0, 0)	0.53	1.1	0.05	0.41	-	-	-	-
ID		0.01 (-0.01, 0.02)	0.31	1.28	0.07	0.39	-	-		
IMD		0.01 (0, 0.02)	0.25	1.1	0.05	0.41	-	-	-	-
GINI-ID		-0.65 (-1.9, 0.6)	0.23	0.85	0.03	0.43	-	-	-	-
GINI-IMD		-0.84 (-2.39, 0.71)	0.29	0.86	0.03	0.43	-	-	-	-
SFI		0 (-0.03, 0.04)	0.76	1.41	0.09	0.38	-	-	-	-
SCI		-0.01 (-0.03, 0)	0.14	0.76	0.03	0.44	-	-	-	-
IDS-BME		0.29 (-1.62, 2.19)	0.77	1.37	0.08	0.39	-	-	-	-
DEN-BME		0.23 (-0.38, 0.85)	0.46	0.87	0.03	0.43	-	-	-	-

For peer review only

Supplementary Table 6 Multi-level modelling of paranoid delusions and global hallucinations. Relevant scores were regressed on predictor variables using multi-level logistic analyses. Significant p values are in bold.

		Persecutory delusions		Global hallucinations	
		Fixed part of the model		Fixed part of the model	
Predictor	Level	Odds ratio (95% CIs)	Wald p value	Odds ratio (95% CIs)	Wald p value
Age		1.03 (1, 1.06)	0.09	0.99 (0.96, 1.02)	0.36
Gender	Female	1.01 (0.59, 1.72)	0.97	0.86 (0.52, 1.44)	0.57
NS-SEC	Managerial	0.22 (0.07, 0.66)	**<0.01**	1.58 (0.51, 4.9)	0.43
	Intermediate	0.91 (0.32, 2.59)	0.86	1 (0.38, 2.59)	1
	Routine	0.75 (0.38, 1.46)	0.39	1.04 (0.55, 2)	0.9
	Student	0.72 (0.36, 1.44)	0.36	2.11 (0.97, 4.63)	0.06

For peer review only

STROBE Statement—Checklist of items that should be included in reports of ***cross-sectional studies***

	Item No	Recommendation
**Title and abstract**	1	(*a*) Indicate the study's design with a commonly used term in the title or the abstract **(Page 1: title)**
		(*b*) Provide in the abstract an informative and balanced summary of what was done and what was found **(Page 2: Abstract)**
**Introduction**		
Background/rationale	2	Explain the scientific background and rationale for the investigation being reported **(Pages 4-5)**
Objectives	3	State specific objectives, including any prespecified hypotheses **(Page 5: lines 23-27; Page 6: lines 1-2)**
**Methods**		
Study design	4	Present key elements of study design early in the paper **(Page 6: line 6)**
Setting	5	Describe the setting, locations, and relevant dates, including periods of recruitment, exposure, follow-up, and data collection **(Page 6: lines 6-13)**
Participants	6	(*a*) Give the eligibility criteria, and the sources and methods of selection of participants **(Page 6: lines 16-22)**
Variables	7	Clearly define all outcomes, exposures, predictors, potential confounders, and effect modifiers **(Page 7 line 5 – Page 9 line 5)**. Give diagnostic criteria, if applicable **(Page 6: lines 16-22)**
Data sources/ measurement	8*	For each variable of interest, give sources of data and details of methods of assessment (measurement). Describe comparability of assessment methods if there is more than one group **(Page 7 line 5 – Page 9 line 5) and (Page 6 line 25 – Page 7 line 2)**
Bias	9	Describe any efforts to address potential sources of bias **(N/a)**
Study size	10	Explain how the study size was arrived at **(N/a)**
Quantitative variables	11	Explain how quantitative variables were handled in the analyses **(Page 8 line 2 – Page 9 line 5; Page 9 line 25 – Page 10 line 3)**. If applicable, describe which groupings were chosen and why
Statistical methods	12	(*a*) Describe all statistical methods, including those used to control for confounding **(Page 9 line 8 – Page 10 line 6)**
		(*b*) Describe any methods used to examine subgroups and interactions **(N/a)**
		(*c*) Explain how missing data were addressed **(Page 10 line 19 – Page 11 line 13)**
		(*d*) If applicable, describe analytical methods taking account of sampling strategy **(N/a)**
		(*e*) Describe any sensitivity analyses **(N/a)**
**Results**		
Participants	13*	(a) Report numbers of individuals at each stage of study—eg numbers potentially eligible, examined for eligibility, confirmed eligible, included in the study, completing follow-up, and analysed (**Unfortunately, information as to the number of potential participants that were evaluated, screened and eventually excluded was not routinely recorded throughout the study. Consequently, we cannot present these data; this limitation has now been noted in the manuscript).**
		(b) Give reasons for non-participation at each stage **(N/a)**
		(c) Consider use of a flow diagram **(N/a)**
Descriptive data	14*	(a) Give characteristics of study participants (eg demographic, clinical, social) and

For peer review only

For peer review only - http://bmjopen.bmj.com/site/about/guidelines.xhtml

		information on exposures and potential confounders **(Page 10 line 19 – Page 11 line 13); see page 12 Table 1 also.**
		(b) Indicate number of participants with missing data for each variable of interest **(Page 10 lines 19 – 27)**
Outcome data	15*	Report numbers of outcome events or summary measures **(Page 12 Table 1)**
Main results	16	(*a*) Give unadjusted estimates and, if applicable, confounder-adjusted estimates and their precision (eg, 95% confidence interval). Make clear which confounders were adjusted for and why they were included **(Page 15 Table 2 and all results)**
		(*b*) Report category boundaries when continuous variables were categorized **(Page 9 line 25 – Page 10 line 3)**
		(*c*) If relevant, consider translating estimates of relative risk into absolute risk for a meaningful time period **(N/a)**
Other analyses	17	Report other analyses done—eg analyses of subgroups and interactions, and sensitivity analyses **(Page 11 lines 7-10)**
**Discussion**		
Key results	18	Summarise key results with reference to study objectives **(Page 16 lines 3-12)**
Limitations	19	Discuss limitations of the study, taking into account sources of potential bias or imprecision. Discuss both direction and magnitude of any potential bias **(Page 18 line 23 – page 20 line 10)**
Interpretation	20	Give a cautious overall interpretation of results considering objectives, limitations, multiplicity of analyses, results from similar studies, and other relevant evidence **(Page 16 line 14 – page 18 line 21)**
Generalisability	21	Discuss the generalisability (external validity) of the study results **(Page 20 lines 12-24)**
**Other information**		
Funding	22	Give the source of funding and the role of the funders for the present study and, if applicable, for the original study on which the present article is based **(Page 3 lines 16-22)**

*Give information separately for exposed and unexposed groups.

Note: An Explanation and Elaboration article discusses each checklist item and gives methodological background and published examples of transparent reporting. The STROBE checklist is best used in conjunction with this article (freely available on the Web sites of PLoS Medicine at http://www.plosmedicine.org/, Annals of Internal Medicine at http://www.annals.org/, and Epidemiology at http://www.epidem.com/). Information on the STROBE Initiative is available at www.strobe-statement.org.

For peer review only
